# Close relatives of Mediterranean endemo-relict hoverflies (Diptera, Syrphidae) in South Africa: Morphological and molecular evidence in the *Merodon melanocerus* subgroup

**DOI:** 10.1371/journal.pone.0200805

**Published:** 2018-07-20

**Authors:** Snežana Radenković, Nevena Veličković, Axel Ssymank, Dragana Obreht Vidaković, Mihajla Djan, Gunilla Ståhls, Sanja Veselić, Ante Vujić

**Affiliations:** 1 Department of Biology and Ecology, University of Novi Sad, Novi Sad, Serbia; 2 Bundesamt für Naturschutz, Bonn, Germany; 3 Zoology Unit, Finnish Museum of Natural History, University of Helsinki, Helsinki, Finland; Helmholtz Zentrum Munchen Deutsches Forschungszentrum fur Umwelt und Gesundheit, GERMANY

## Abstract

An ongoing study of the genus *Merodon* Meigen, 1803 in the Republic of South Africa (RSA) has revealed the existence of new species related to *M*. *melanocerus* Bezzi, 1915. The *M*. *melanocerus* subgroup belongs to the Afrotropical lineage of the *M*. *desuturinus* group. Revision of all available material from museums and detailed analyses of newly -collected specimens from our own expeditions to RSA resulted in delimitation of five species: *M*. *capensis* Hurkmans sp. n., *M*. *commutabilis* Radenković et Vujić sp. n., *M*. *drakonis* Vujić et Radenković sp. n., *M*. *flavocerus* Hurkmans sp. n. and *M*. *melanocerus*. In addition to classical morphological characters, sequences of the mitochondrial COI gene are provided for four related taxa. Results of molecular phylogenetic analyses supports monophyly of the *M*. *desuturinus* group and confirmed delimitation between species. Links between Palaearctic and Afrotropical faunas of this group, as well as possible evolutionary paths, are discussed. Based on phylogenetic analyses, four lineages (putative subgenera) have been recognized within the genus *Merodon*; besides the three previously established ones, *albifrons*+*desuturinus*, *aureus* (sensu lato) and *avidus*-*nigritarsis*, one new lineage named *natans* is distinguished.

## Introduction

The phytophagous hoverfly genus *Merodon* Meigen (Diptera: Syrphidae: Eristalinae: Merodontini) is distributed over the Palaearctic and Ethiopian regions and comprises more than 160 species [1]. The highest diversity of this genus is recorded in the Mediterranean region, which has been associated with the high variety of bulb plants that are larval hosts [2, 3, 4]. The species that has received the most attention is the narcissus bulb fly, *Merodon equestris* Fabricius, 1794, which is considered a pest in flower bulb cultivation. This is the only representative of the genus in the Nearctic region, where it was probably inadvertently introduced.

The most comprehensive revision of the *Merodon* genus was done by Hurkmans [5], who assessed 61 species and classified them into 11 groups (*alagoezicus*, *alexeji*, *avidus*, *clavipes*, *crassifemoris*, *elegans*, *longicornis*, *nigritarsis*, *pruni*, *tarsatus* and *vandergooti*) in the first part of the monograph. The second part of the monograph has never been published, but handed to Ante Vujić in manuscript form. Later, a series of papers dealing with particular groups of species, such as *aureus*, *nanus*, *nigritarsis* and *ruficornis*, have appeared [6–12]. The *Merodon* fauna of the Balkan Peninsula, Aegean Islands, Turkey and the Iberian Peninsula are the most comprehensively explored in Europe [1, 13–21], contrasting sharply with the hoverfly fauna of the Afrotropical region. Although this region harbours about 600 hoverfly species, representatives of the genus *Merodon* are very rare. Less than ten species have been recorded, and most data are from South Africa [22]. Intriguingly, the Cape region is the most diverse in bulb plants of the African continent, with ca. 1200 species [23].

The *Merodon desuturinus* species group is very interesting because it represents an important link between the Palaearctic and Afrotropical faunas, and may reveal the possible origin of the genus. Vujić, Šimić and Radenković [24] described the Balkan endemic *Merodon desuturinus* Vujić, Šimić et Radenković, 1995 from high mountains and, later, Marcos-García et al. [14] discovered another related species, *M*. *cabanerensis* Marcos-García, Vujić et Mengual, 2007 from Spain that was also confirmed in Morocco [25]. Vujić et al. [25] described one additional species *M*. *neolydicus* Vujić, 2018, distributed in the Eastern Mediterranean, and redescribed *M*. *murorum* (Fabricius, 1794) from North-West Africa. These authors, as well as Milankov, Ståhls and Vujić [26], highlighted diagnostic characters for the *desuturinus* group and emphasized that the African species *M*. *cuthbertsoni* Curran, 1939 and *M*. *planifacies* Bezzi, 1915, also clearly belonged to this group.

A basic phylogeny of the genus was established by Mengual et al. [13] who defined four well-supported clades based on analysis of COI sequences from 17 Iberian species: the *desuturinus*, *albifrons*, *nigritarsis* (*avidus*) and *aureus* groups. Milankov et al. [26] analysed genetic relationships among populations of *M*. *desuturinus* and taxa from the *aureus*, *avidus* and *ruficornis* groups from the Balkan Peninsula and observed low genetic variability in populations of *M*. *desuturinus*. Furthermore, Milankov et al. [26] showed that *M*. *desuturinus* represents an evolutionarily independent lineage among *Merodon* taxa and that an integrative taxonomic approach should be applied for further insights into the evolutionary history and phylogenetic position of the *M*. *desuturinus* group. Šašić et al. [12] merged the *albifrons* and *desuturinus* groups into one clade according to the results of Vujić et al. [9]. This is in agreement with close relationships between these two groups previously described by Milankov et al. [26], who also highlighted the *ruficornis* group (belonging to the *albifrons* clade) as being the closest one to *desuturinus*.

A recent barcoding study of Afrotropical hoverflies [27] stressed the importance of using molecular data to assist morphological identification of species and ecological studies, since identification of Afrotropical hoverflies is challenging. Nuclear rDNA (ITS2, 28S, 18S) and mtDNA sequences are commonly used genetic markers in population genetic studies, taxonomy, systematics and conservation genetics. The mtDNA cytochrome c oxidase I (COI) gene has repeatedly been used as a useful taxonomic tool for species delimitation in the family Syrphidae [1, 9, 12, 12, 19, 20, 28]. Integrative taxonomic studies have used both the 3’- and 5’-ends of COI. The latter is widely regarded as a “barcoding” sequence and it represents one of the most used genetic markers for species identification [29–33]. The 3’-end of COI also constitutes a useful taxonomic tool in studies of the Syrphidae [9, 34, 35]. According to Jordaens et al. [27], there are few COI barcodes of Afrotropical hoverflies in public databases (BOLD Identification System and GenBank). For South Africa, we found 23 records of hoverfly COI sequences available in GenBank (in a search of the nucleotide collection with keywords ‘Syrphidae’ and ‘South Africa’ on June 27th, 2018), with no representative of the genus *Merodon*. Jordaens et al. [27] provided a COI barcode reference database for 98 Afrotropical hoverfly species, mostly from western Africa, but did not include species from the genus *Merodon*. They encouraged expansion of the database to other genera and geographical areas since molecular data not only assist species identification, but also provide a basis to pinpoint taxa that need further taxonomic studies, help identify recent introductions, and can be useful for linking conspecific males and females having discordant morphologies and larvae with adults, thereby enhancing our understanding of the ecology of particular species.

Specifically, here we present an ongoing study of the genus *Merodon* in the Republic of South Africa (RSA), as well as a detailed analysis of available material from museums and private collections, that has revealed the existence of new species of the *desuturinus* group, related to *M*. *melanocerus* Bezzi, 1915, native to southeastern RSA. In addition to classical morphological characters, we obtained sequences of the 3’ and 5’ regions of the mitochondrial COI gene for 23 specimens of the *M*. *melanocerus* subgroup and combined the data with that from 27 other *Merodon* taxa. Records noted here represent the first detailed characterisation of *Merodon* species in South Africa based on classical morphological and molecular data, and constitute a contribution towards a molecular database of Afrotropical Syrphidae.

## Material and methods

### Morphological analysis

**Fig 1:**
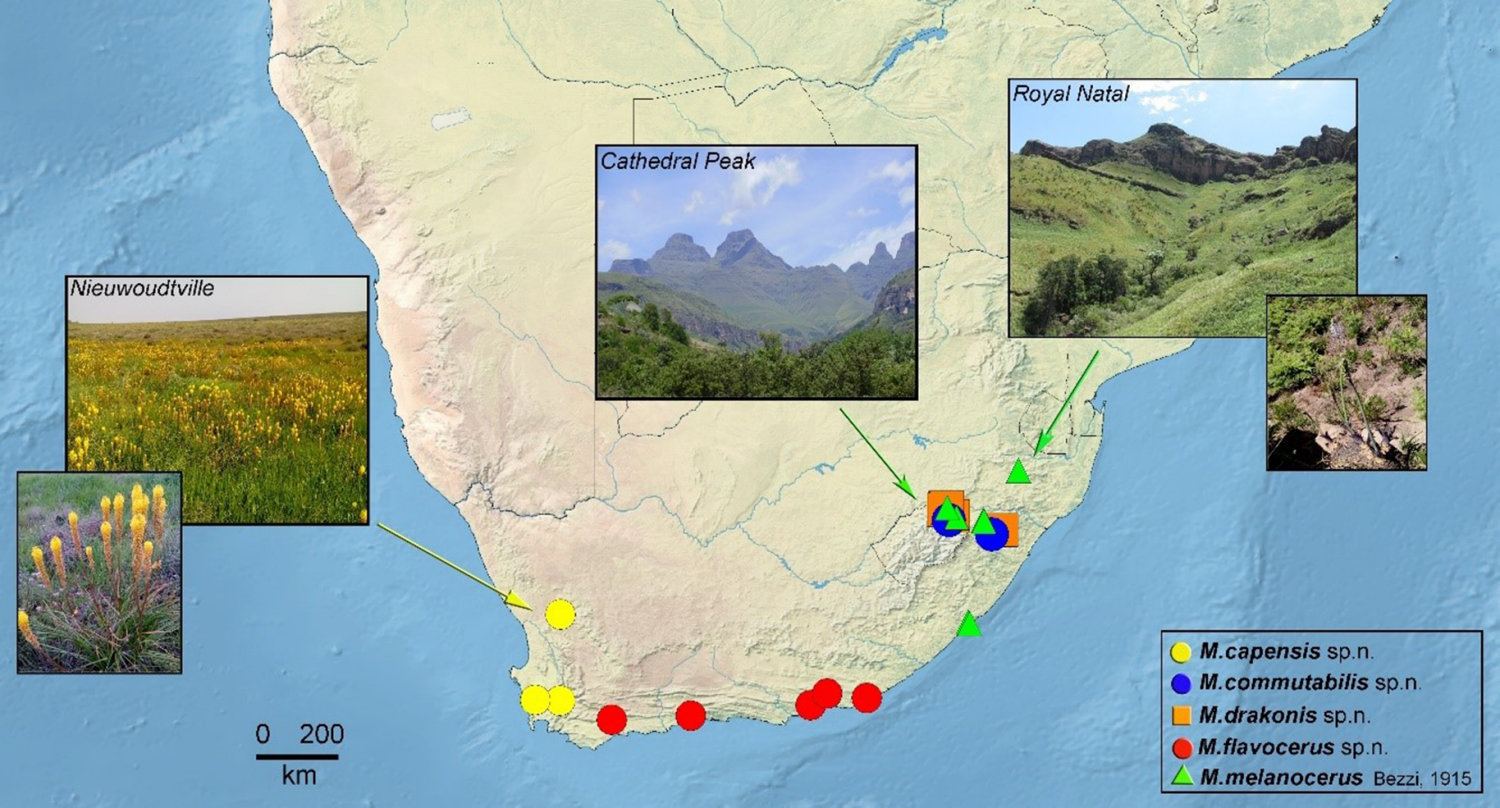
Distribution of the sample localities for the *Merodon melanocerus* subgroup.

Permits for insect collecting were obtained from Ezemwelo KwaZulu-Natal province: permits OP 3754/2015 and OP 4603/2016, Eastern Cape province: CRO 15/16CR and CRO 02/16CR. This revision is based on the examination of *Merodon* specimens collected in RSA during expeditions between 2011 and 2016 [collectors: Jelena Ačanski (BioSense, Serbia), Celeste Pérez Bañón (CEUA, Spain), Ximo Mengual (ZFMK, Germany), Marija Miličić (FSUNS, Serbia), Snežana Radenković (FSUNS, Serbia), Santos Rojo (CEUA, Spain), Axel Ssymank (A.S.coll., Germany), Gunilla https://www.researchgate.net/profile/Gunilla_StahlsStåhls (MZH, Finland), Nevena Veličković (FSUNS, Serbia) and Ante Vujić (FSUNS, Serbia). In addition, we studied all the available Afrotropical material previously cited in the bibliography and unpublished material deposited in the museums, universities and private collections listed below. Type specimens of all *Merodon* species described from the Afrotropics were also studied. The following acronyms for museums and entomological collections containing studied material are used in the text: BMNH—Natural History Museum, London, UK; CEUA—Colección Entomológica Universidad de Alicante, Spain; FSUNS—Faculty of Sciences, Department of Biology and Ecology, University of Novi Sad, Serbia; MZH—Finnish Museum of Natural History, University of Helsinki, Finland; NMNL—National Museum of Natural History Naturalis, Leiden, Netherlands; NMSA—Natal Museum, Pietermaritzburg, Republic of South Africa; ZFMK—Zoological Research Museum Alexander Koenig, Bonn, Germany; ZMUC—Zoological Museum, University of Copenhagen, Denmark; A.S.coll.—Axel Ssymank collection, Germany; M.B.coll.—Miroslav Bartak collection, Czech Republic, M.R.coll.—Menno Reemer collection, Netherlands. Data on the geographic distribution of analyzed species were processed in DivaGis (v7.5) [36] and presented on Fig 1. The characters used follow the terminology established by Thompson [37], Doczkal and Pape [38] and those relating to male genitalia are those employed by Marcos-García et al. [14]. Colour characters are described from dry-mounted specimens. The male genitalia were extracted from dry specimens previously relaxed in a closed pot with a high level of humidity, by using an insect pin with a hooked tip. They were cleared by boiling in warm 10% potassium hydroxide (KOH) for 3–5 minutes. Acetic acid was then used to neutralize the KOH during 5 seconds. Genitalia were stored in microvials containing glycerol. Specimen measurements were taken in dorsal view with a micrometer and are presented as ranges. Body length was measured from the lunula to the end of the abdomen, and wing length from the base of the tegula to the wing apex. Drawings were made with an FSA 25 PE drawing tube attached to a binocular microscope Leica MZ16. Photos were made with a Leica DFC320 camera connected to a personal computer. After photographing, CombineZ software [39] was used in order to create composite image with an extended depth of field, created from the in- focus areas of each image. Photo of pile was taken with a JEOL JSM 6460LV scanning electron microscope (SEM) operated at 20 kV.

A short description is provided for each new species, including figures of adult morphology. Diagnoses comprise accounts of unique characters relative to the group, lineage, subgroup and species considered here, and also combinations of characters that enable taxa to be distinguished and recognized. Keys are also provided to enable identification of adults. The type material was examined by Ante Vujić.

### Nomenclatural acts

The electronic edition of this article conforms to the requirements of the amended International Code of Zoological Nomenclature, and hence the new names contained herein are available under that Code from the electronic edition of this article. This published work and the nomenclatural acts it contains have been registered in ZooBank, the online registration system for the ICZN. The ZooBank LSIDs (Life Science Identifiers) can be resolved and the associated information viewed through any standard web browser by appending the LSID to the prefix “http://zoobank.org/”. The LSID for this publication is: urn:lsid:zoobank.org:pub: 9DD25199-47F6-412E-B89A-D4A1D9505161. The electronic edition of this work was published in a journal with an ISSN, and has been archived and is available from the following digital repositories: PubMed Central, LOCKSS.

### Molecular analysis

#### DNA extraction

Total genomic DNA was extracted using 1 to 3 legs from 23 dry, pinned specimens of the *M*. *melanocerus* subgroup following the procedure described by Chen et al. [40]. Genomic DNA vouchers were accordingly labelled and conserved at the Faculty of Sciences, Department of Biology and Ecology, University of Novi Sad (FSUNS).

#### PCR amplification and sequencing

The mtDNA COI 3’ fragments were amplified using the forward primer C1-J-2183 (5’-CAA CAT TTATTT TGA TTT TTT GG-3’) (alias JERRY) and reverse primer TL2-N-3014 (5’-TCC AAT GCA CTA ATC TGC CAT ATT 3’) (alias PAT) [41]. Additionally, the Folmer fragment or ‘barcode fragment’ of the 5' region of COI was amplified using standard polymerase chain reaction protocols with the forward primer LCO (5'-GCTCAACAAATCATAAAGATATTGG-3') and reverse primer HCO (5'-TAAACTTCAGGGTGACCAAAAAATCA-3') [42]. The reaction mix contained 1x reaction buffer (Thermo Scientific), 2.5 mM MgCl_2_, 0.1 mM of each nucleotide, 2 pmol of each primer, 1U Taq polymerase (Thermo Scientific), and approximately 50 ng template DNA in a total volume of 25μl. Amplification was performed using the following conditions: 95°C for 2 min; 29 cycles of 94°C for 30s each, 49°C (for 3’ COI) and 50°C (for 5’ COI) for 30 s; 72°C for 2 min; with the final extension at 72°C for 8 min. The PCR products were purified using ExoSAP (Thermo Scientific) following the manufacturer’s recommendations. Sequencing was conducted on an ABI3730xl Genetic Analyzer (Applied Biosystems, Folmer City, CA, USA).

#### Phylogenetic analysis

Obtained sequences of the 3’ and 5’ regions of COI were aligned using the Clustal W algorithm [43] implemented in BioEdit [44], and final adjustments were performed manually. In order to establish the systematic position of the *M*. *melanocerus* subgroup, our phylogenetic analyses also included several species belonging to the *M*. *desuturinus* group, *Merodon* species from the Palaearctic region representing the main lineages following Mengual et al. [13], and six species as outgroups (see S1 Table for accession numbers of examined species and outgroups).

For construction of phylogenetic trees, we removed identical sequences using DAMBE v.5 [45]. The Maximum Likelihood (ML) tree was constructed using MEGA 7 [46] under the general time-reversible (GTR) evolutionary model using a discrete Gamma distribution with five rate categories and by assuming that a certain fraction of sites are evolutionarily invariable as it was shown as the best evolutionary model for the generated dataset (as estimated in MEGA 7). Branch support was estimated with 100 non-parametric bootstrap replicates. Bayesian phylogenetic analyses (BI) were also carried out using the same evolutionary model as for ML tree, as priors in MrBayes ver.3.2 [47]. Two independent runs of four Markov chain Monte Carlo (MCMC) permutations were performed for 20,000,000 generations with sampling every 100 generations. Tracer v1.5 [48] was used to summarise the Bayesian analysis and to inspect the validity of the burn-in fraction applied. The first 25% of the sampled iterations/generations were discarded as burn-in, and 50% consensus trees were computed using FigTree v1.4.0 [49]. Additionally, a phylogenetic analysis, as inferred by Maximum Parsimony (MP) using NONA [50], was conducted with the aid of Winclada [51] using the heuristic search algorithm with 1000 random addition replicates (mult*1000), holding 100 trees per round (hold/100), max trees set to 100 000 and applying tree-bisection-reconnection (TBR) branch swapping. Nodal support for the resulting consensus tree was assessed using non-parametric bootstrapping with 1000 replicates. The tree was rooted with *Microdon bidens*.

## Results

### Systematic position and diagnostic characters of the *Merodon melanocerus* subgroup

**Fig 2:**
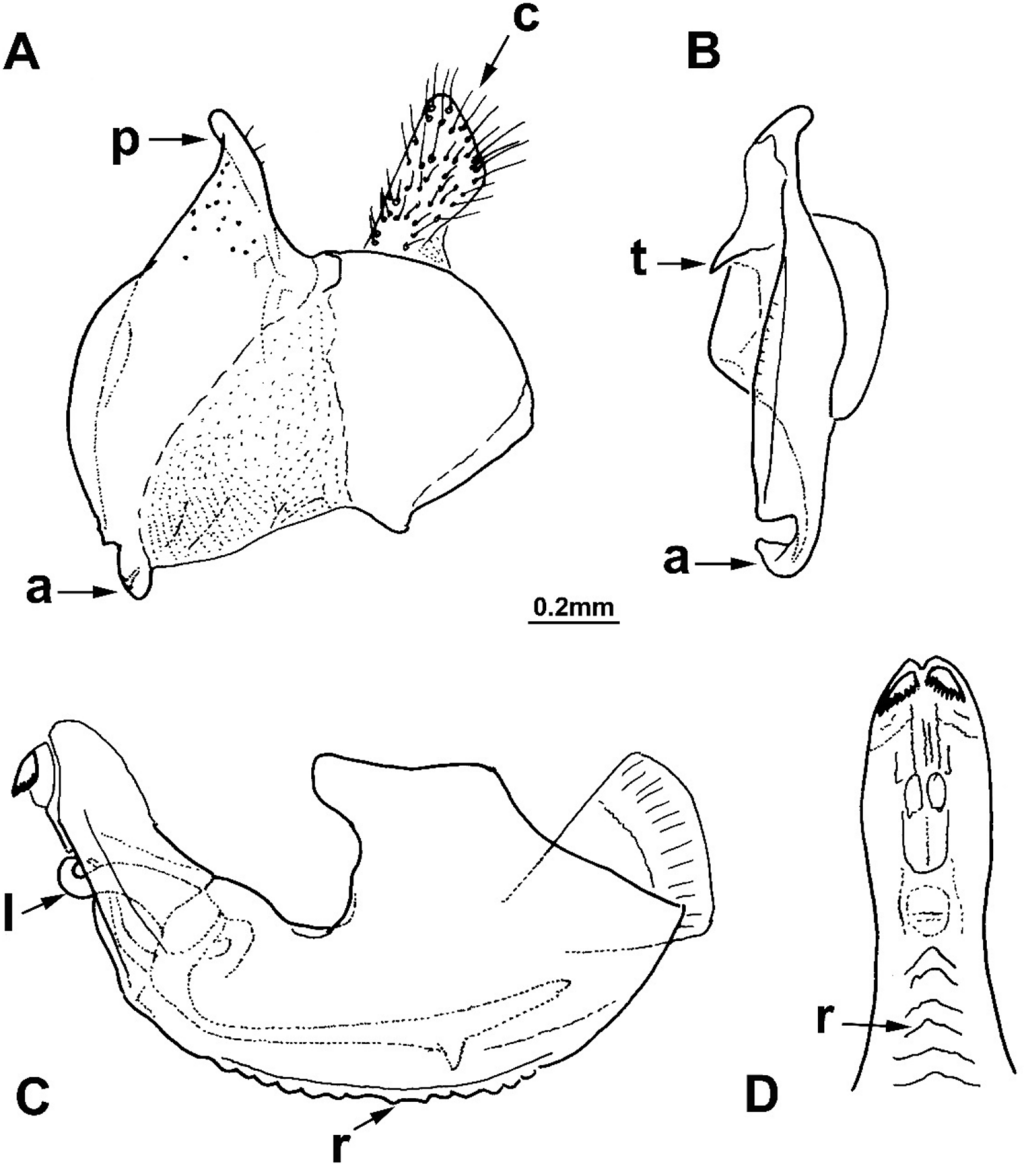
*Merodon capensis* sp. n., male genitalia (holotype). (A) epandrium, lateral view; (B) surstyle lobe, ventral view; (C) hypandrium, lateral view; (D) apical part of the hypandrium, ventral view; (a) anterior lobe of surstyle; (t) inner thorn on median part of surstyle; (p) posterior lobe of surstyle; (c) cercus; (l) lateral sclerite of aedeagus; (r) ventral ridge of theca.

**Fig 3:**
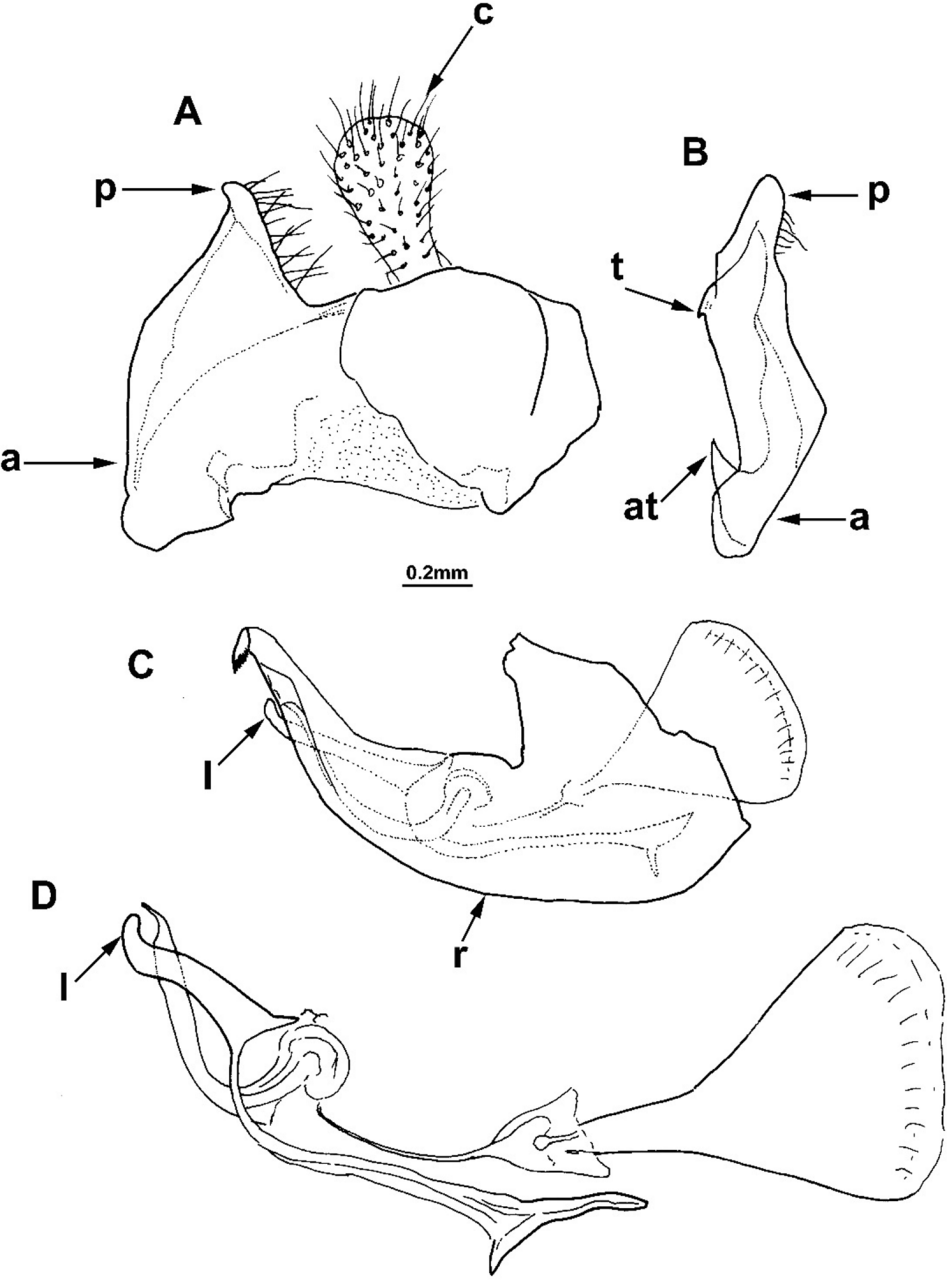
*Merodon melanocerus* Bezzi, 1915, male genitalia (RSA). (A) epandrium, lateral view; (B) surstyle lobe, ventral view; (C) hypandrium, lateral view; (D) aedeagus with accessory structures, lateral view; (a) anterior lobe of surstyle; (at) inner thorn on anterior lobe of surstyle; (p) posterior lobe of surstyle; (t) inner thorns on median part of surstyle; (c) cercus; (l) lateral sclerite of aedeagus; (r) ventral ridge of theca.

**Fig 4:**
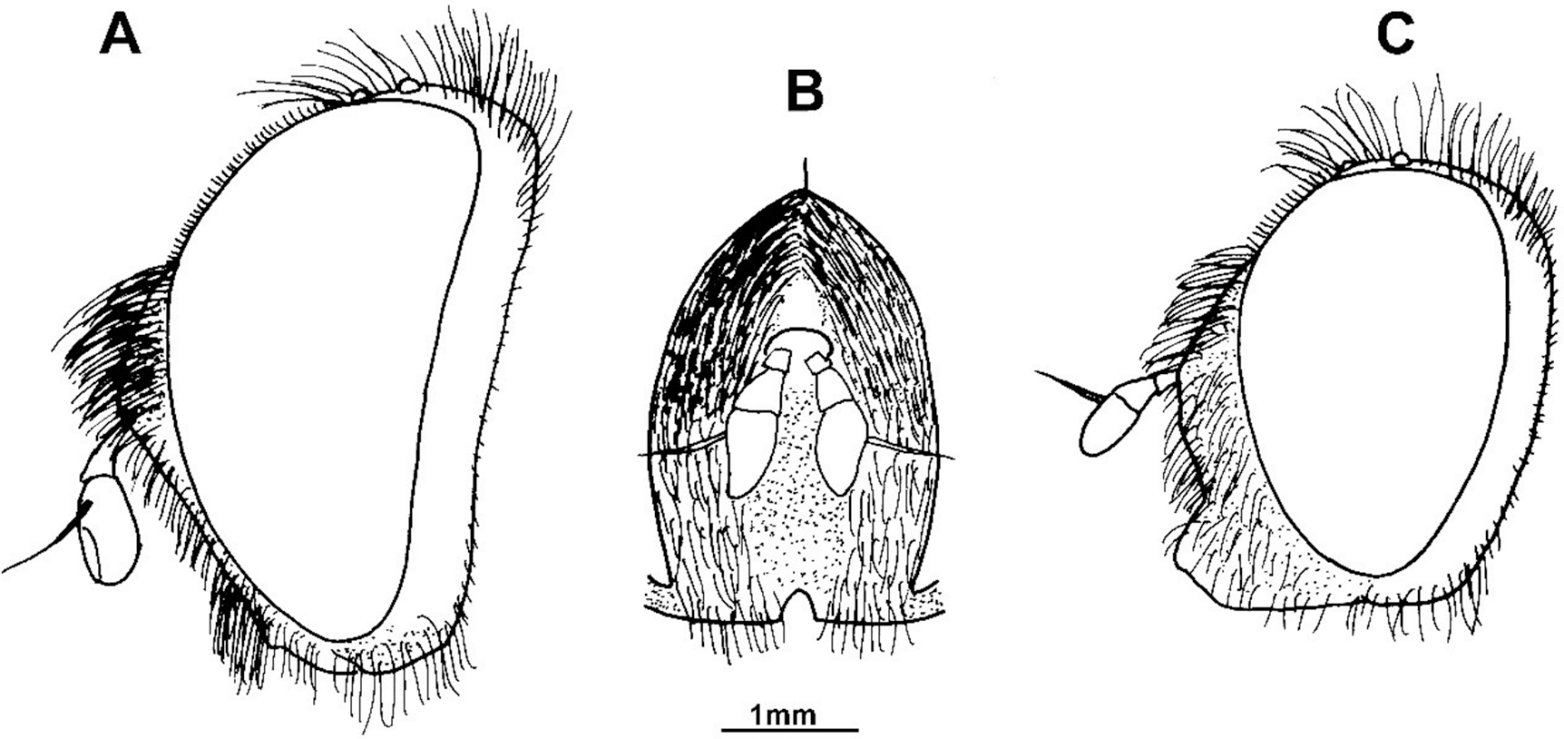
Head, males. (A, B) *Merodon planifacies* Bezzi, 1915 (holotype): (A) lateral view; (B) face, anterior view. (C) *Merodon desuturinus* Vujić, Šimić et Radenković, 1995 (Kopaonik, Serbia), lateral view.

The *Merodon melanocerus* subgroup, defined here based on morphological characteristics, belongs to the *M*. *desuturinus* group sensu Mengual et al. [13] and is closely related to the *albifrons* group [13]. The *M*. *desuturinus* group is characterized by the following adult morphological characters: posterior surface of mesocoxa with pile; anterior lobe of surstyle of male genitalia with curved distal prolongation (as in Fig 2B: a); and the specific shape of the lateral sclerite of the aedeagus (which is the main synapomorphic character for the group) (as in Fig 3D: l) [24, 25, 26]. This group contains a Palaearctic and an Afrotropical lineages. The main morphological diagnostic character that separates these two lineages is the presence of a dense and strong yellow-to-red brush of pile on the metatrochanter in Afrotropical species, which is lacking in Palaearctic taxa. In addition to *M*. *desuturinus*, the Palaearctic lineage includes three more species: *M*. *cabanerensis*, *M*. *murorum* and *M*. *neolydicus*. The Afrotropical lineage comprises four known taxa: *M*. *cuthbertsoni*, *M*. *melanocerus*, *M*. *planifacies* and *M*. *stevensoni* Curran, 1939. New studies have revealed cryptic species within the taxon *M*. *planifacies* and new species related to *M*. *melanocerus*. The *M*. *planifacies* and the *M*. *stevensoni* share a clear apomorphic character, i.e., a reduced oral margin covered by microtrichia (Fig 4A and 4B) and together represent a subgroup named *M*. *planifacies* [25]. Of the remaining species in the group, *M*. *cuthbertsoni* is very close to *M*. *desuturinus* according to morphological characters, and treated together with Mediterranean representatives of the group [25]. Looking at distributions, members of the *M*. *planifacies* subgroup are endemics distributed in Western, Central and Southern Africa, contrary to the species of the *melanocerus* group whose ranges are restricted to Southern Africa. The species *M*. *cuthbertsoni* is endemic, only known from Zimbabwe.

Based on our results we provide a revision of taxa related to the species *M*. *melanocerus* that cluster together in a subgroup of the *M*. *desuturinus* group, all having the following additional diagnostic characters: protruded and shiny oral margin and face; thorax black (except in *M*. *flavocerus* sp. n. with orange postpronotum and postalar calli); tergite 2 often with a pair of orange lateral spots (except in *M*. *capensis* sp. n. and *M*. *commutabilis* sp. n.); punctuation in general less dense, especially on lateral sides of tergites; females can be distinguished from related species by long and outstanding white pile on sternites and on the lateral sides of tergites, especially when compared with the length of adpressed pile on the central parts of the tergites.

### General description of *Merodon melanocerus* subgroup

**Fig 5:**
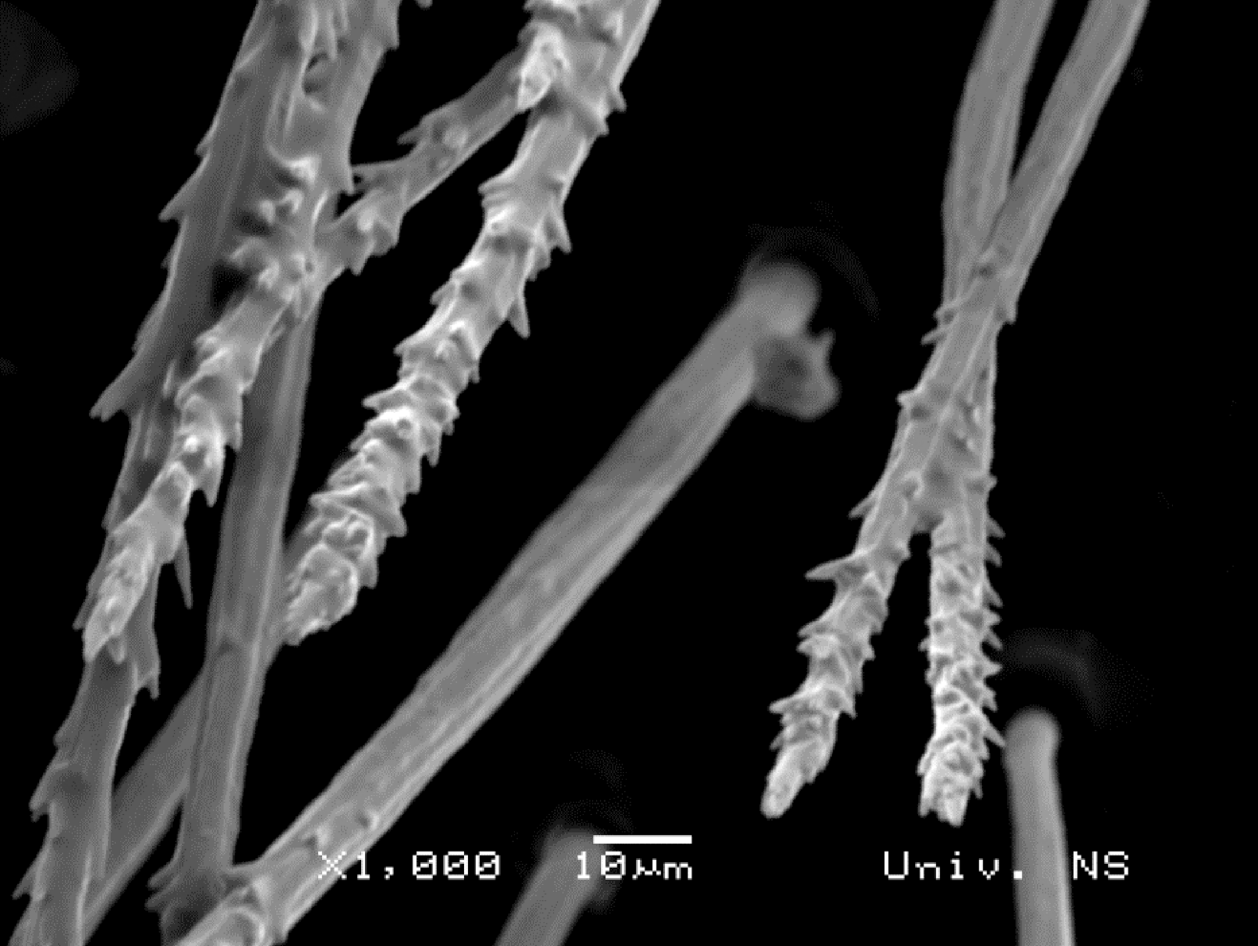
Pile on ocellar triangle of *Merodon drakonis* sp. n. (SEM).

**Fig 6:**
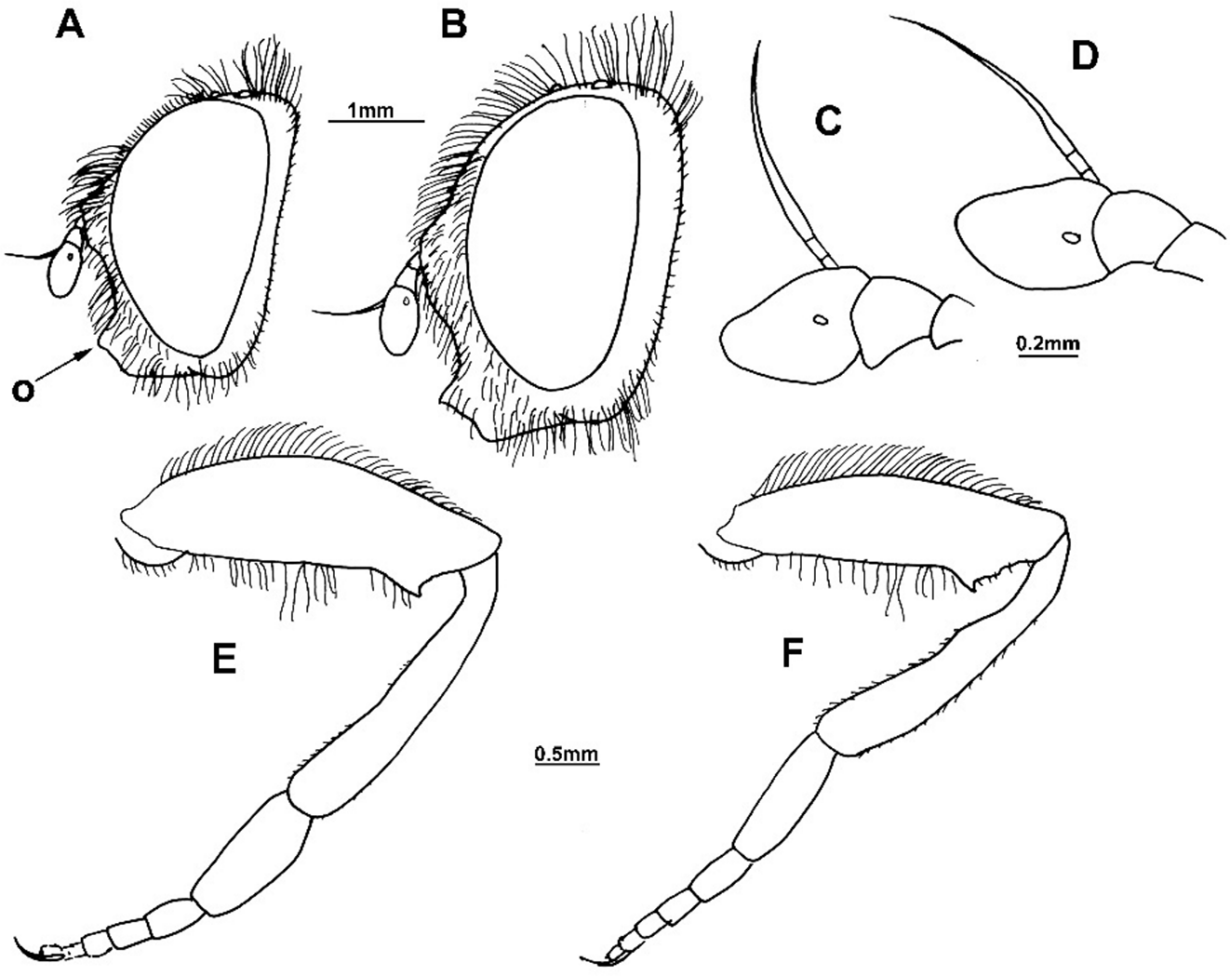
*Merodon capensis* sp. n. (male holotype RSA, Cape Province, female paratype RSA, Northern Cape, Hantam Botanical Garden). (A) head of male, lateral view: (o) oral margin; (B) head of female, lateral view; (C) antenna of male, lateral view; (D) antenna of female, lateral view; (E) hind leg of male, anterior view; (F) hind leg of female, anterior view.

**Fig 7:**
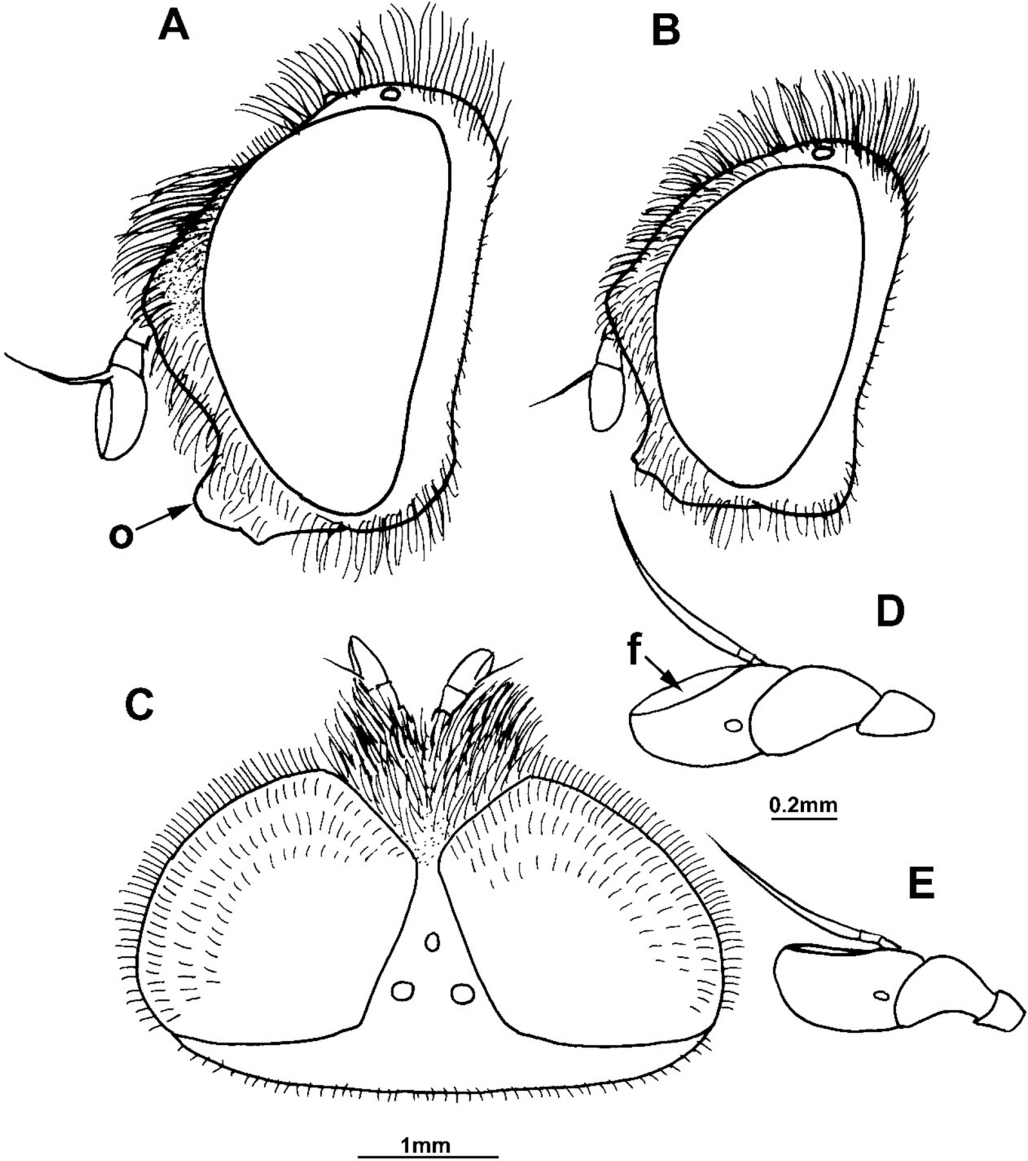
*Merodon flavocerus* sp. n. (paratype, RSA). (A) head of male, lateral view: (o) oral margin; (B) head of female, lateral view; (C) head of male, dorsal view; (D) antenna of male, lateral view: (f) fossette; (E) antenna of female, lateral view.

Male. Body pile generally branched (Fig 5). Head (as in Fig 6A): Antenna (as in Fig 6C) usually dark brown; basoflagellomere generally short, as long as broad (except in *M*. *flavocerus* sp. n. where it is light brown and longer, Fig 7D), concave dorsally, with acute apex; arista light brown to dark brown, thickened basally, 1.5–2 times longer than basoflagellomere, covered with dense brown microtrichia. Face shiny black, with narrow white microtrichose stripe along eye margin, often missing in lower third (except in *M*. *flavocerus*, where it is present all along eye margin), covered with long whitish-yellow pile, except on median bare stripe that occupies 1/4 width of face. Oral margin shiny black, well protruded (as in Figs 6A and 7A). Frons black, often with bronze shine and indistinct microtrichia that, at level of face, run in a narrow line along the eye margin. Vertical triangle isosceles (as in Fig 7C), black (brown-red in *M*. *flavocerus*), shiny except for anterior end with microtrichia; predominantly covered with long, black, thick pile (very thick in *M*. *commutabilis* sp. n. and *M*. *drakonis* sp. n.), except at posterior end with pale yellow pile (in *M*. *melanocerus* sp. n. pale pile also present on anterior end). Eye pile dense, long as scape, often pale yellow, but can be darker dorsally (in *M*. *commutabilis* black and thick dorsally). Occiput covered with whitish-yellow pile; dorsally with metallic, bluish or bronze lustre; white microtrichia start from upper eye corner as a narrow line dorsally, becoming dense and wide laterally and ventrally, occupying lower 2/3 of occiput.

Thorax: Scutum and scutellum black with bronze lustre (in *M*. *flavocerus*, postpronotum and posterior rim of scutellum pale yellow); presence of microtrichia variable (from well-developed in *M*. *drakonis* to absent in *M*. *capensis*); covered with relatively long (as long as, or a little longer than basoflagellomere), dense, erect, more or less branched, usually yellow pile (in *M*. *capensis* and *M*. *commutabilis* mixed with black pile). Pleurae often covered with gray-green microtrichia (lacking in *M*. *flavocerus*) and the following parts with long yellow pile: anterior part of proepimeron, posterior part of anterior anepisternum, most of the posterior anepisternum except anterior end, antero-ventral and postero-dorsal part of katepisternum, anepimeron, metasternum; katatergite with dense, erect, short, yellowish or light brown pile. Wing hyaline, with dense microtrichia and light brown to dark brown veins. Calypter yellow. Halter with brown pedicel and yellow to brown capitulum. Legs usually dark brown to black (light brown in *M*. *flavocerus*), except paler knees, and sometimes paler base and apex of tibiae (like in *M*. *melanocerus* and *M*. *flavocerus*); colour of tarsi varies. Metatrochanter without process, covered with brush of yellow-to-orange, dense, strong pile. Metafemur moderately thickened and straight or slightly curved. Metatibia with apical, inconspicuous antero-ventral spur and indication of postero-ventral spur. Pile on legs predominantly yellow, except some short black pile dorsally on tarsi (and sometimes in *M*. *capensis* and *M*. *commutabilis* partly present on femora).

Abdomen: Black with bronze reflections, slightly tapering, as long as mesonotum. Tergites 2–4 black with more or less distinct white transverse band of microtrichia interrupted in the middle (can be connected on tergite 4); tergite 2 with a pair of orange antero-lateral spots (lacking in *M*. *capensis* and *M*. *commutabilis*, which have dark tergites) or areas covered with long, dense, erect, yellow pile; pilosity on lateral sides of tergites long, erect and whitish, but adpressed on central parts, white on mictrotichose transversal bands, posterior 2/3 of tergite 4 and also on hind margin of tergites 2–3 of most species (except in *M*. *capensis*), otherwise black. Sternites shiny, dark brown (except yellow in *M*. *flavocerus*), covered with very long pale yellow pile.

**Fig 8:**
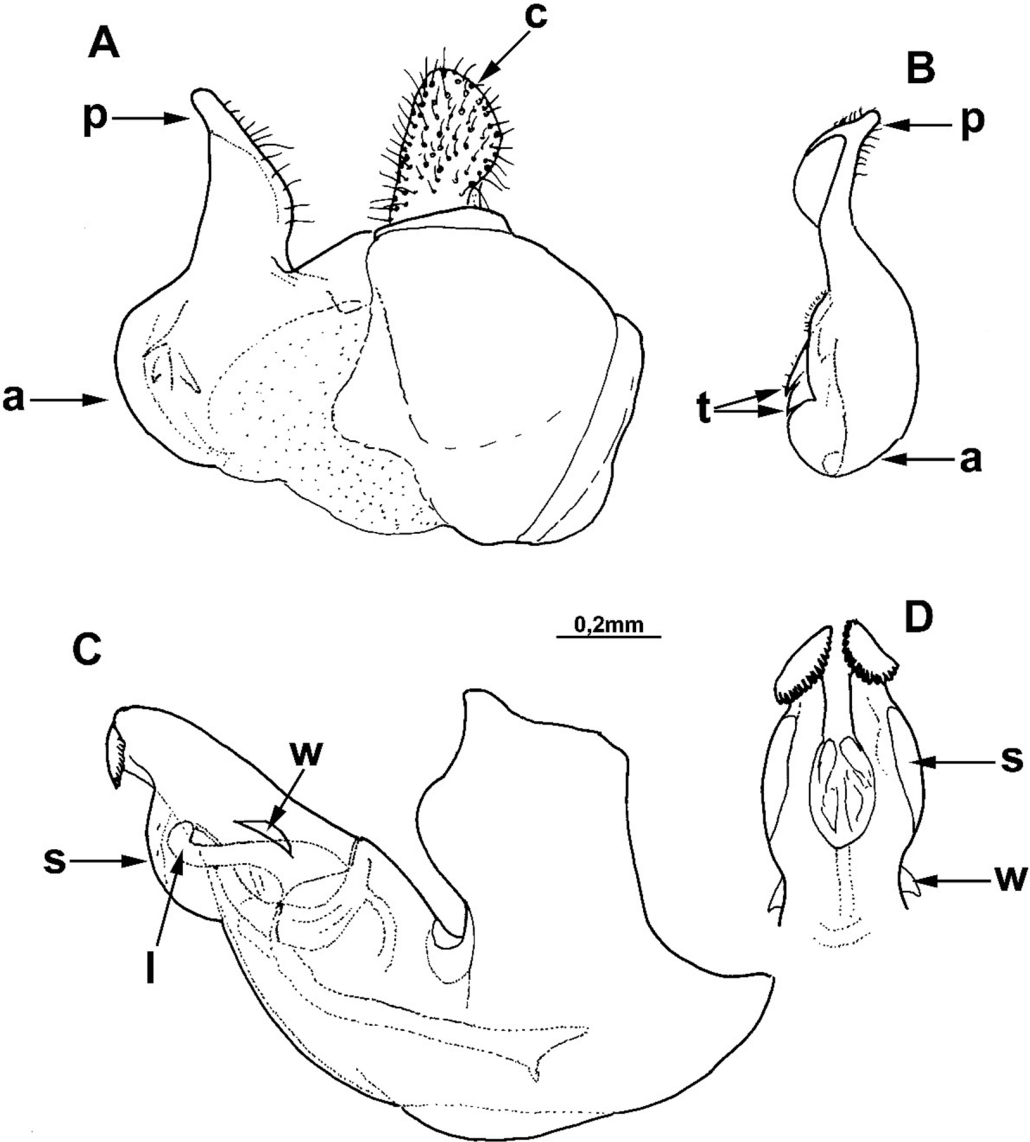
*Merodon commutabilis* sp. n., male genitalia (holotype, KwaZulu-Natal, RSA). (A) epandrium, lateral view; (B) surstyle lobe, ventral view; (C) hypandrium, lateral view; (D) apical part of the hypandrium, ventral view; (a) anterior lobe of surstyle; (p) posterior lobe of surstyle; (t) inner thorns on median part of surstyle; (c) cercus; (l) lateral sclerite of aedeagus; (s) subapical lamella of theca; (w) lateral wings of theca.

Male genitalia: Posterior lobe of surstyle triangular, usually pointed apically (as in Fig 2A: p); ventral margin of anterior lobe of surstyle straight (as in Fig 3A: a) or convex (as in Fig 8A: a); anterior lobe of surstyle bent inwards (as in Fig 2B: a); median part of surstyle with one or two inner thorns (as in Fig 8B: t); cercus elongated (as in Figs 2A and 3A: c). Hypandrium with broad theca (as in Fig 2C). Lateral sclerite of aedeagus narrow, gradually tapering, with the tip curved downwards (as in Fig 3D: l).

**Fig 9:**
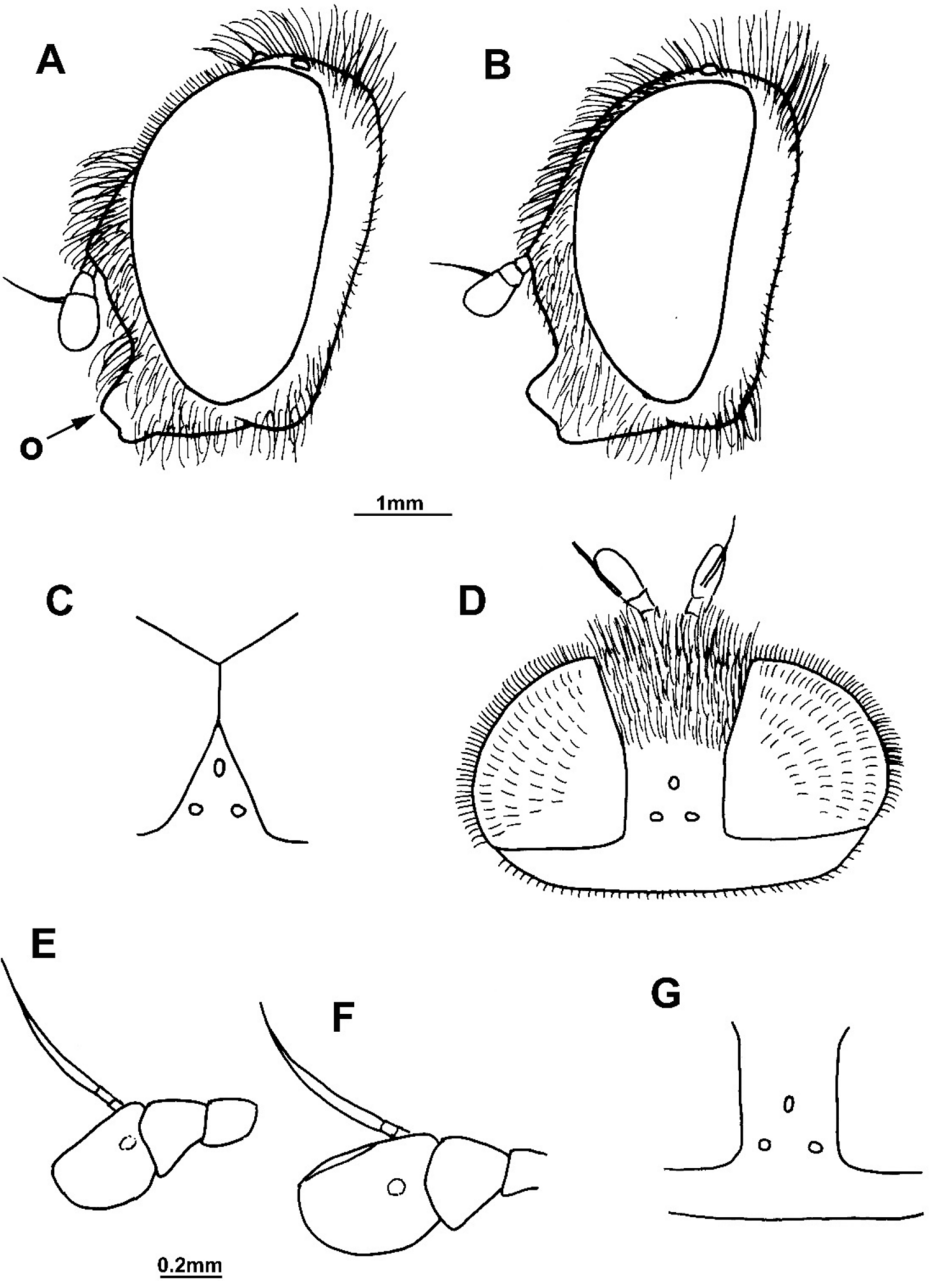
(A-F) ***Merodon melanocerus* Bezzi, 1915** (RSA): (A) head of male, lateral view: (o) oral margin; (B) head of female, lateral view; (C) vertex of male, dorsal view; (D) head of female, dorsal view; (E) antenna of male, lateral view; (F) antenna of female, lateral view. (G) ***Merodon drakonis* sp. n.** (RSA, Drakensberg Mountains): vertex of female, dorsal view.

Female. Similar to the male except for normal sexual dimorphism (as in Fig 9D).

Length: medium-sized species, body 10–12 mm, wing 6–8 mm (n = 65).

Biology. Flight period is mainly from June to December, although some specimens were also recorded in February. Adults were observed resting on leaves of low vegetation (grass, bulb plants), and in some cases feeding on flowers of *Senecio* spp. Larvae are unknown.

#### *Merodon capensis* Hurkmans sp. n. urn:lsid:zoobank.org:act:D04600C6-D629-4456-B5F1-3986C2378983 (Figs 2 and 6)

Holotype. ♂, RSA, Northern Cape, Nieuwoudtville, Hantam National Botanical Garden, 31^o^ 23' 51" S, 19^o^ 08' 24" E, 11 September 2012, J. & A. Londt (sweep net), (NMSA) (GUN7 = 08899).

Paratypes: RSA, Cape. 30km NE Wellington, Bainskloof Pass, 1♀, 27 September 1979, J. Londt (sweep net) (ZMUC) (04330); RSA, Capland, Stellenbosch, 1♂, 25 December 1925, H. Brauns (sweep net), det. P.H. van Doesburg as *Lampetia nasica* Bezzi, (NMNL) (LML-05-6); RSA, Capland, Stellenbosch, 2mm, 25 September 1925, H. Brauns (sweep net), (NMSA); RSA, Capland, Stellenbosch, 1♂, 25 December 1925, H. Brauns (sweep net), det. P.H. van Doesburg as *Lampetia nasica* Bezzi, (NMSA)(48544); RSA, Northern Cape, Nieuwoudtville, Hantam National Botanical Garden, 31^o^ 23' 51" S, 19^o^ 08' 24" E, 1♂, 1♀, 11 September 2012, J. & A. Londt, (NMSA) (08898, 08900).

Range (Fig 1). Endemic species for the Cape Region in RSA. *Flight period*: from September to December.

Etymology. The name *capensis* is derived from the word ‘Cape’ referring to the Cape Province in South Africa, the type area of the species.

**Fig 10:**
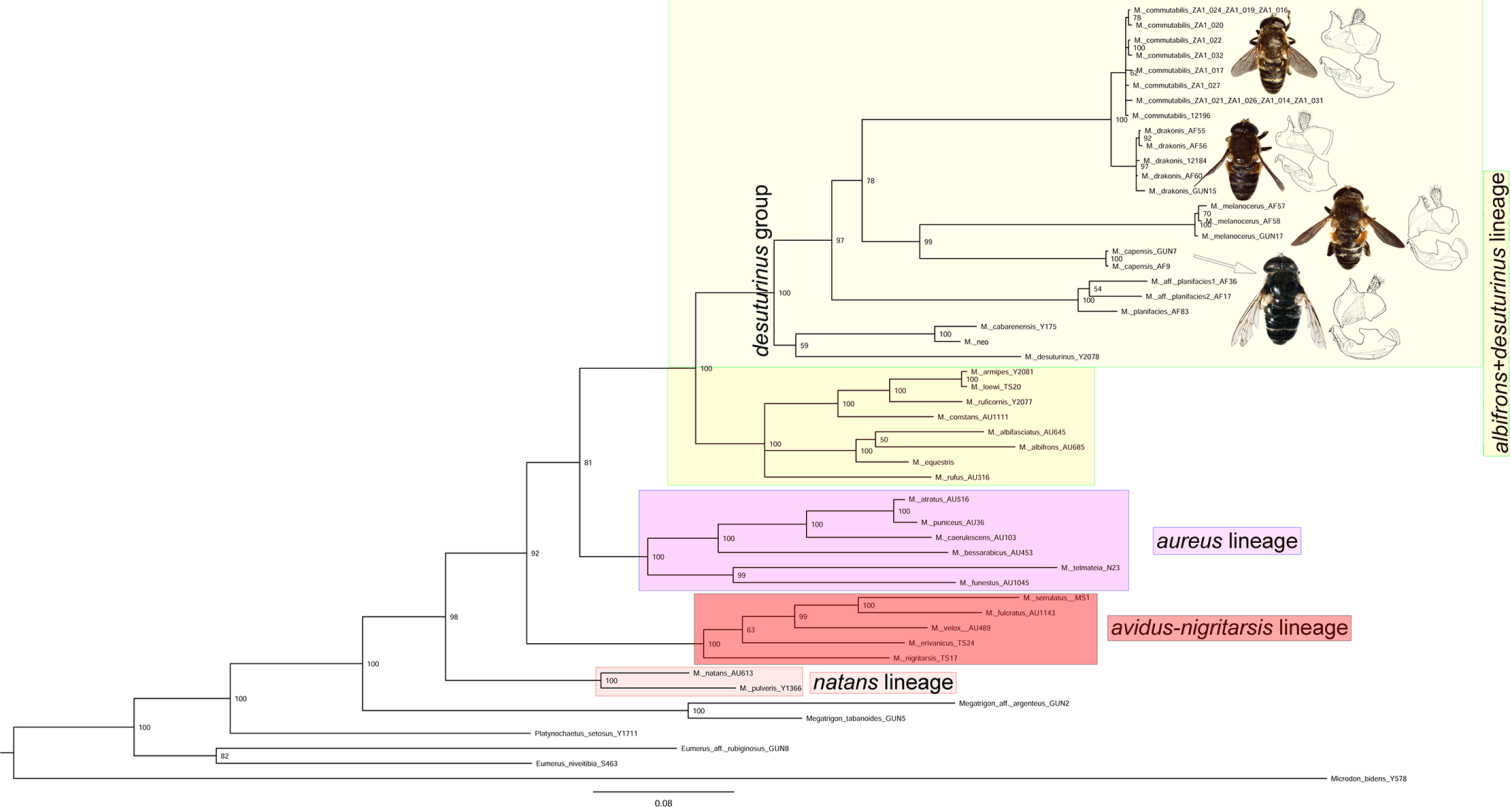
Bayesian interference tree based on analysis of combined 3'- and 5'- fragments of the COI gene. Numbers on nodes depict Bayesian posterior probabilities. The scale bar indicates expected number of changes by site. Four lineages observed in genus *Merodon* are marked on the tree. For species that belong to *Merodon melanocerus* subgroup, photos of adult specimens and male genitalia are presented.

Diagnosis. Scutum between wing bases with stripe of black pile, usually without microtrichia (except in some specimens present on anterior margin, but without longitudinal microtrichose stripes or microtrichia on transverse suture). Тergites black; transversal stripes of microtrichia on tergites 2–4 narrow, less than 1/8 of their length, in some males can be absent on tergite 4. Tibiae and tarsi partly pale-yellow-brown, especially basal two or three tarsal segments on pro- and mesolegs. Thecal ridge folded in male genitalia (Fig 2C and 2D: r). Female frons mostly black pilose. According to structure of male genitalia and genetic data, the most closely related species is *M*. *melanocerus* (Fig 10), from which it can be easily distinguished by lack of orange lateral spots on tergite 2 and the absence of a large inner thorn on the anterior lobe of surstyle of the male genitalia, as well as the wider hypandrium, besides previously mentioned diagnostic characters. Most similar taxon is *M*. *commutabilis*, which is also a black species but has completely black legs (contrary to *M*. *capensis*) and a conspicuously narrower posterior lobe of surstyle of the male genitalia.

Description. Male. Head (Fig 6A): Antenna (Fig 6C) brown-to-black, basoflagellomere 1.1–1.2 times as long as wide, 1.4 times longer than pedicel; arista dark brown, 1.5 times longer than basoflagellomere. Vertical triangle isosceles, twice as long as eye contiguity. Ocellar triangle equilateral. Eye contiguity about 10–12 ommatidia long. Eye pile mostly pale yellow.

Thorax: Scutum and scutellum black with bronze lustre (usually without microtrichia except in some specimens on anterior margin of scutum), covered with dense, erect, gray-whitish or yellow pile, except for stripe of black pile between wing bases. Wing hyaline with dark-brown veins, and dense microtrichia except for cell bc, and above and below spurious vein in proximal 2/3 of cell br, with reduced microtrichia. Femora and tibiae brown-black, except usually with paler knees and base of tibiae; tarsi dark brown dorsally (except usually pale on basal two or three tarsal segments on pro- and mesolegs), orange-to-light brown ventrally. Pile on legs yellow, but in some specimens antero-dorsal part of pro- and mesofemora and apical 1/3 of metafemur can have short black pile, besides to black pile on dorsal surface of metatarsus. Metafemur (Fig 6E) slightly curved.

Abdomen: Tergites 2–4 black with more or less distinct white transverse band of microtrichia interrupted in the middle (in some specimens lacking on tergite 4); pile on tergites erect and whitish-yellow on lateral sides, but tergites 2–4 medially with adpressed black pile, except white pile on microtrichose bands.

Male genitalia (Fig 2): Anterior lobe of surstyle bent inwards (Fig 2B: a), with ventral margin slightly convex (Fig 2A); median part of surstyle with one inner thorn (Fig 2B: t); posterior lobe of surstyle wide, triangular, pointed apically (Fig 2A: p). Hypandrium wide, with folded thecal ridge (Fig 2C and 2D: r).

Female. Similar to the male except for normal sexual dimorphism (Fig 6B, 6D and 6F) and shiny frons without microtrichia (exceptionally with very narrow line of microtrichia along eye margin), mostly covered with black pile.

#### *Merodon commutabilis* Radenković et Vujić sp. n. urn:lsid:zoobank.org:act:6707360D-736D-495A-8CAE-07F4C29EA65E (Fig 8)

Holotype. ♂, RSA, KwaZulu-Natal, Howick, near Currys Post, 29° 21' 43.9" S, 30° 5' 54.9" E, 18 October 2015, A. Vujić (sweep net), (NMSA) (ZA1_014).

Paratypes: RSA, KwaZulu-Natal, Howick, near Currys Post, 29° 21' 43.9" S, 30° 5' 54.9" E, 17♂♂, 3♀♀, 18 October 2015, A. Vujić, J. Ačanski & M. Miličić (sweep net), (CEUA, FSUNS, NMSA & MZH) (ZA1_013, ZA1_015–032, ZA1_215); RSA, KwaZulu-Natal, Van Reenen, 1♂, Drakensberg, December 1926, R. E. Turner (sweep net), (BMNH); RSA, KwaZulu-Natal, near Howick, meadow near exit 125 of N3 to Balgowan, 1260m, 29^o^ 21' 45.9" S, 30^o^ 05' 52.5" E, 2♂♂, 2♀♀, 18 October 2015, X. Mengual (sweep net), (ZFMK) (12193–12196).

Range (Fig 1). Distributed in KwaZulu-Natal Province in RSA. *Flight period*: October to December.

Etymology. The name *commutabilis* is a Latin word meaning changeable/variable, referring to the great variability of pile colour on the scutum.

Diagnosis. Black species with white narrow transversal microtrichose bands on tergites 2–4, similar to *Merodon capensis* sp. n., but clearly different in male genitalia structure (hypandrium with unfolded theca, but with distinct subapical lamellas and lateral wings; epandrium with much narrower posterior lobe of surstyle and more convex ventral margin of anterior lobe of surstyle), presence of microtrichose stripes on scutum (in *M*. *capensis* absent or reduced microtrichia on anterior margin), and black legs (partly pale in *M*. *capensis*). Based on the structure of the male genitalia and genetic data, it is closest to *M*. *drakonis* (Fig 10), from which it can be distinguished by the lack of large, distinct, orange lateral spots on tergite 2 and subtle differences in male genitalic characters (hypandrium and epandrium shorter, posterior lobe of surstyle wider). Body pile very thick and branched, especially visible on vertical triangle and scutum.

Description. Male. Head: Antenna dark-brown, basoflagellomere 1.1 times as long as wide, 1.2 times longer than pedicel, concave dorsally, apex acute; arista about twice as long as basoflagellomere. Vertical triangle isosceles, 2.5 times longer than eye contiguity, covered with long black pile. Ocellar triangle isosceles. Eye contiguity about 10 ommatidia long. Eye pile mostly pale yellow, except for thick black pile on dorsal 1/3.

Thorax: Scutum and scutellum black with bronze lustre, and three more or less developed white microtrichose longitudinal stripes (from distinct three stripes long as 2/3 length of scutum to specimens with indistinct medial stripe reaching level of transverse suture and reduced submedial stripes) and microtrichia on transverse suture; colour of pile on scutum extremely variable, from all pale, whitish or yellowish to specimens with a stripe of black pile between wing bases or almost completely black pilosee in some specimens. Wing hyaline, with dense microtrichia and brownish veins. Halter brown. Legs dark brown to black, exceptionally with paler ventral side of tarsi. Metatrochanter with very distinct brush of hairs. Legs predominantly covered with pale yellow pile, except short black pile on dorsal and antero-dorsal parts of pro- and mesofemora, and in some specimens also on distal end of metafemur. Metafemur slightly thickened and curved.

Abdomen: Tergites 2–4 black, with distinct white transverse band of microtrichia interrupted in the middle; tergite 2 usually completely black, exceptionally with small, vague, orange or brown spot in front of mictrotrichose band, never reaching lateral margin of tergite; pile on tergites mainly erect and yellow on lateral sides, but tergites 2–4 medially with black adpressed pile.

Male genitalia (Fig 8): Ventral margin of anterior lobe of surstyle convex (Fig 8A: a); median part of surstyle with two inner thorns (Fig 8B: t); posterior lobe of surstyle elongated, pointed apically (Fig 8A: p). Hypandrium broad, with smooth thecal ridge (Fig 8C), well-developed subapical lamellas (Fig 8C and 8D: s) and lateral wings (Fig 8C and 8D: w).

Female. Similar to the male except for normal sexual dimorphism and shiny frons with microtrichia only present along eye margin, covered with mixed pale yellow and black pile.

#### *Merodon drakonis* Vujić et Radenković sp. n. urn:lsid:zoobank.org:act:44EFC9F3-E610-4F60-A1E6-6AC3F0F75495 (Figs 5, 9G and 11)

Holotype. ♂, RSA, Royal Natal NP, Thendele, 28^o^ 41' 18" S, 28^o^ 55' 50.7" E, 1–6 December 2012, A. Vujić (sweep net), (NMSA) (G2137 = GUN15).

**Fig 11:**
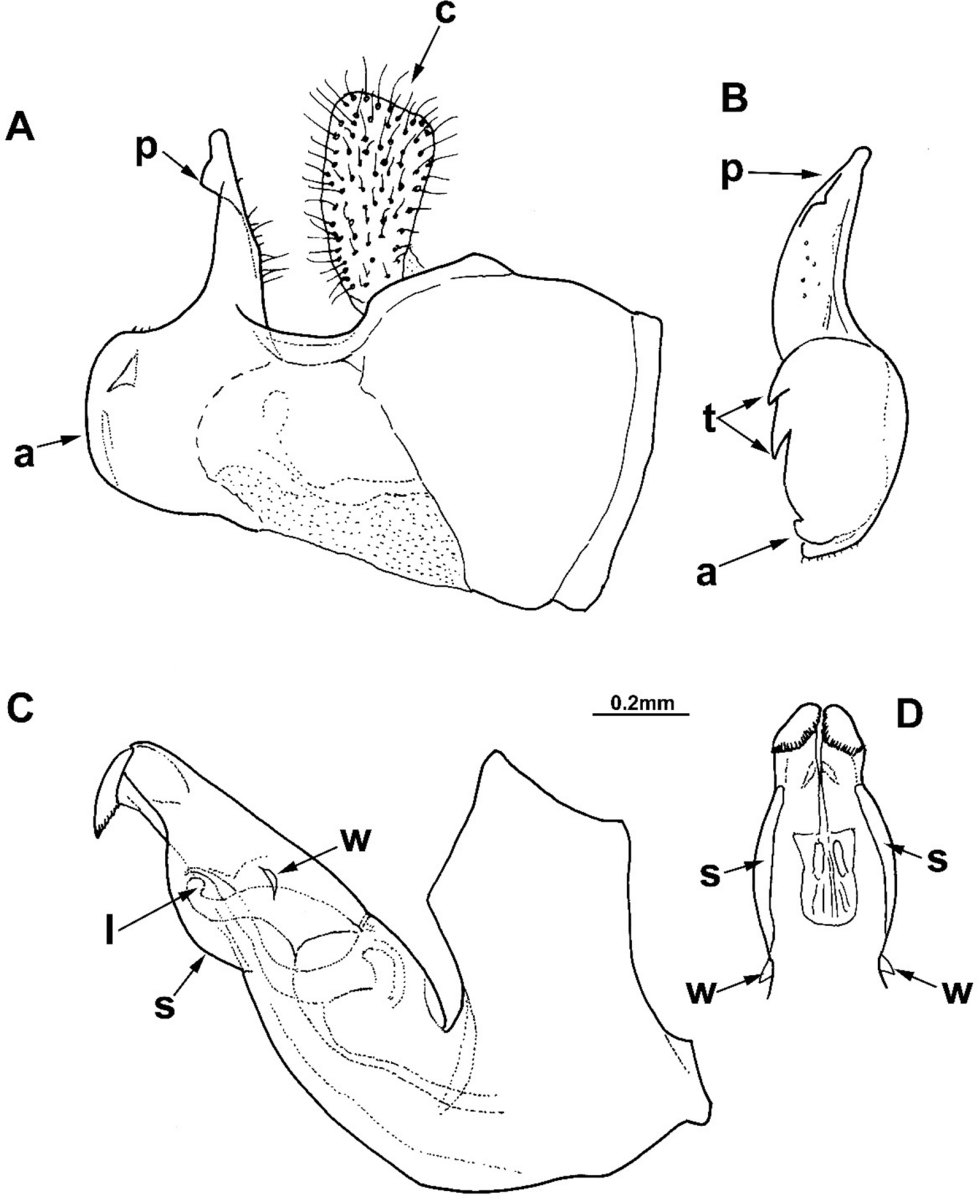
*Merodon drakonis* sp. n., male genitalia (holotype RSA, Drakensberg Mountains). (A) epandrium, lateral view; (B) surstyle lobe, ventral view; (C) hypandrium, lateral view; (D) apical part of the hypandrium, ventral view; (a) anterior lobe of surstyle; (p) posterior lobe of surstyle; (t) inner thorns on median part of surstyle; (c) cercus; (l) lateral sclerite of aedeagus; (s) subapical lamella of theca; (w) lateral wings of theca.

Paratypes: RSA, Royal Natal NP, Thendele, 28^o^ 41' 18" S, 28^o^ 55' 50.7" E, 7♂♂, 2♀♀, 1–6 December 2012, C. Pérez-Bañón, G. Ståhls & A. Vujić (sweep net), (CEUA, FSUNS, NMSA & MZH) (G2133, G2135, AF55, G2174—G2178); Thendele, 28^o^ 43' 0" S, 28^o^ 55' 0" E, 1♂, 3 December 2012, S. Radenković (sweep net), (FSUNS) (G2125); RSA, Royal Natal NP, Crack, 28^o^ 41' 18" S, 28^o^ 55' 50.7" E, 3♂♂, 8–9 December 2012, S. Rojo (sweep net), (CEUA, FSUNS & NMSA) (05778, AF56, G2212); RSA, Royal Natal NP, Sentinel, 28° 44' 23.9"S, 28° 53' 2" E, 1♂, 2♀♀, 8 December 2012, A. Vujić (sweep net), (FSUNS) (G2222, AF60, G2227); RSA, Natal, Benvie farm, 29^o^ 15' 30" S, 30^o^ 20' 40" E, mixed *Podocarpus* forest edge, 1♂, 4 December 1987, J. Londt & A. Seymour (sweep net), (NMSA) (50843); RSA, Royal Natal NP, Gorge Car Park, 28^o^ 42' 48.5" S, 28^o^ 56' 06.0" E, 1530m, 2mm, 3 December 2012, A. Ssymank (sweep net), (A.S.coll.) (AB 10); RSA, Cathedral Peak, Rainbow Gorge, 1♂, November 2011, Vujić A. (sweep net), (FSUNS) (G0878); RSA, KwaZulu-Natal, near Howick, meadow near exit 125 of N3 to Balgowan, 1260m, 29^o^ 21' 45.9" S, 30^o^ 05' 52.5" E, 1♂, 18 October 2015, X. Mengual (sweep net), (ZFMK) (12184); RSA, Drakensberg Mountains, Sunday Falls, -28° 40' 16.3" S, 28° 57' 11" E, 11 ♂♂, 1♀, 22 February 2016, S. Radenković, N. Veličković & A. Vujić (sweep net), (NMSA & FSUNS) (ZA2_137- ZA2_148).

Range (Fig 1). Endemic species to Drakensberg Mountains in RSA. Sympatric and synchronic with *M*. *commutabilis*. *Flight period*: February and November/December.

Etymology. The name *drakonis* is derived from the name of the type area, i.e. Drakensberg ("mountains of dragons"), the main mountain range in South Africa.

Diagnosis. This species is very similar in appearance to *Merodon melanocerus* (short basoflagellomere, almost as long as wide; tergite 2 with reddish-orange lateral spots). The main differences between males of these two species are the shapes of the genitalia: posterior lobe of surstyle narrow, straight, pointed, with a small apical ridge (Fig 11A: p) (in *M*. *melanocerus* triangular, without apical ridge, Fig 3A: p); ventral margin of anterior lobe of surstyle convex (Fig 11A: a) (in *M*. *melanocerus* almost straight, Fig 3A: a). For females, the distance between the posterior ocellus and upper eye corner in *M*. *drakonis* sp. n. is less than the distance between ocelli (Fig 9G) (in *M*. *melanocerus*, this distance is larger, Fig 9D). Based on the structure of male genitalia and genetic data (Fig 10), it is most closely related to *M*. *commutabilis* (see diagnosis of *M*. *commutabilis*).

Description. Male. Head: Antenna dark-brown, basoflagellomere 1.1–1.2 times as long as wide, 1.1–1.3 times longer than pedicel; arista about twice as long as basoflagellomere. Vertical triangle isosceles, 2.5 times longer than eye contiguity. Ocellar triangle slightly isosceles. Eye contiguity about 10 ommatidia long. Eye pile mostly pale yellow, except for some black pile on apical 1/6.

Thorax: Scutum with well-developed whitish microtrichia: anteriorly, laterally on transverse suture and on intraalar area with longitudinal stripe extending from transverse suture to the level of posterior end of postalar callus, medially with three longitudinal stripes long as 2/3 length of scutum. Microtrichia of pleurae conspicuous, the most developed on katepisternum, gray-green in colour. Halter brown. Legs dark brown-black, except slightly paler on knees and ventral side of tarsi. Metafemur slightly thickened and curved. Legs with pale yellow pile.

Abdomen: Tergites 2–4 with distinct white transverse band of microtrichia interrupted in the middle; tergite 2 with large, orange antero-lateral spots; pile on tergites mainly erect and yellow on lateral sides, but tergites 2–4 medially with black adpressed pile. Sternites can be paler, especially posterior half of sternite 1, almost all of sternite 2 and partly sternite 3 light brown; shiny, except in some specimens the anterior margin of sternite 4 has microtrichia.

Male genitalia (Fig 11): Epandrium and hypandrium generally prolate (Fig 11A and 11C). Ventral margin of anterior lobe of surstyle convex (Fig 11A: a); posterior lobe of surstyle elongated, apically pointed, with small ridge (Fig 11A: p); median part of surstyle with two inner thorns (Fig 11B: t). Hypandrium with smooth thecal ridge (Fig 11C), distinct subapical lamellas (Fig 11C and 11D: s) and lateral wings (Fig 11C and 11D: w).

Female. Similar to the male except for normal sexual dimorphism (Fig 9G) and for the following characteristics: frons with transversal striae, shiny in the center; laterally, beside microtrichose line along eye margin, also with triangular microtrichose areas.

#### *Merodon flavocerus* Hurkmans sp. n. urn:lsid:zoobank.org:act:2BFDB16C-95AF-4B38-B07B-AA4AFF69343A (Figs 7, 12A and 12B)

Holotype. ♂, RSA, Cape Province, 15 km SE Kirkwood, 4 November 1978, R. Miller & J. Londt (sweep net), (NMSA).

Paratypes: RSA, George, Cape Colony, 1♂, 25 December 1922, 1♂, 25 September 1922, (NMNL), 1 October 1920, 1♀, (NMSA), Dr Brauns (sweep net), det. v. Doesburg as *Lampetia melanocera* Bezzi); RSA, Bontebok NP, Western Cape, 1♀, 13–15 October 1993, F. Koch (sweep net), (M.B.coll.); RSA, Pt. Elizabeth, 1♂, 28 February 1919, H. K. Munro (sweep net), det. v. Doesburg as *Lampetia melanocera* Bezzi, (NMSA) (50556); RSA, Cape Province, Kleinemonde, 1♂, 11 September 1959, D. J. Greathead (sweep net), (NMNL).

Range (Fig 1). Species restricted to Cape Province in RSA. *Flight period*: February and September to November.

Etymology. The epithet *flavocerus* had already been affixed to the holotype by F. M. Hull, who did not publish the species description; this epithet has been retained by Hurkmans in his unpublished manuscript. It was probably given by Hull in reference to the yellowish antennae. It is to be treated as a noun.

**Fig 12:**
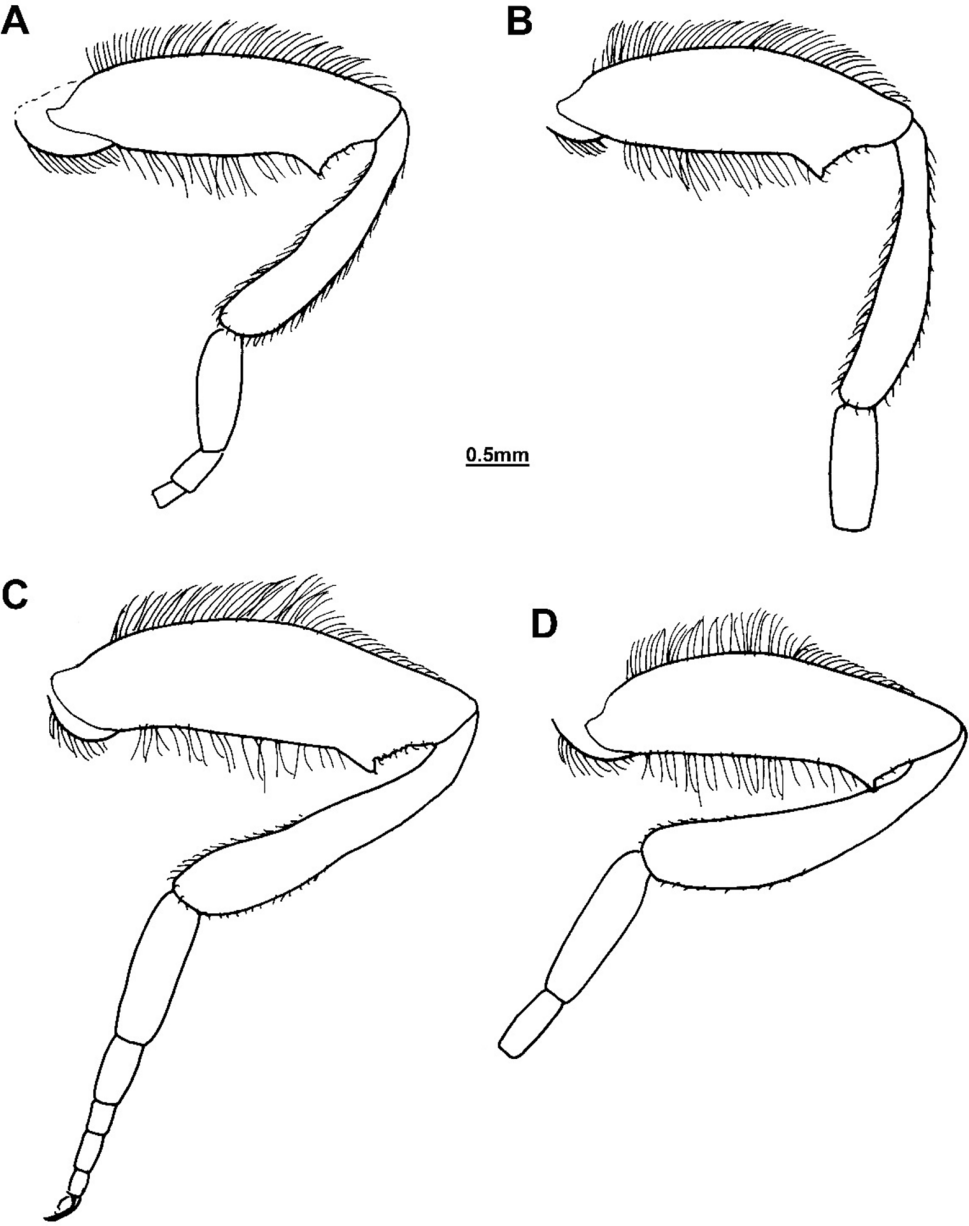
Hind legs—anterior view. (A, B) *Merodon flavocerus* sp. n. (paratype, RSA); (C, D) *Merodon melanocerus* Bezzi, 1915 (RSA); (A, C) male; (B, D) female.

**Fig 13:**
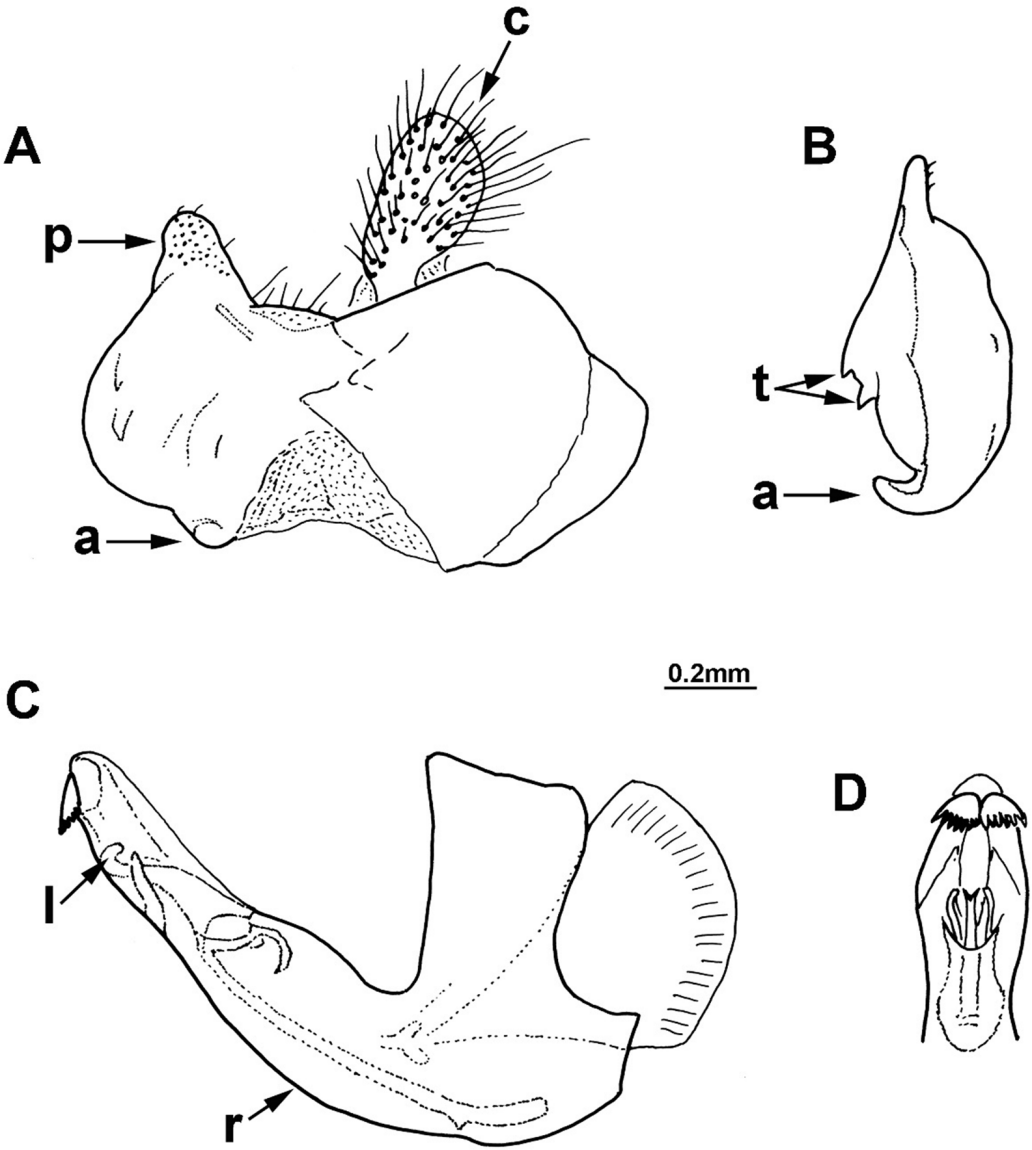
***Merodon flavocerus* sp. n., male genitalia** (paratype, RSA); (A) epandrium, lateral view; (B) surstyle lobe, ventral view; (C) hypandrium, lateral view; (D) apical part of the hypandrium, ventral view; (a) anterior lobe of surstyle; (p) posterior lobe of surstyle; (t) inner thorns on median part of surstyle; (c) cercus; (l) lateral sclerite of aedeagus; (r) ventral ridge of theca.

Diagnosis. Basoflagellomere, orange-brown, elongated 1.5–2.0 times as long as wide (Fig 7D and 7E); metafemur straight, not curved (Fig 12A and 12B); katepisternum shiny; tergites 3–4 with reddish transversal stripes. In males, eyes dichoptic (Fig 7C); posterior lobe of surstyle short, broad and triangular (Fig 13A: p).

Description. Male. Head (Fig 7A and 7C): Antenna (Fig 7D) orange-brown, basoflagellomere 1.8–2.0 times as long as wide, 1.5 times longer than pedicel, with wide fossette occupying whole dorsal surface (Fig 7D: f); arista: second and third flagellomeres yellow, fourth flagellomere light brown basally and dark brown apically, 1.5 times longer than basoflagellomere. Face shiny black, except narrow white microtrichose line along entire eye margin. Vertical triangle (Fig 7C) light brown, shiny red-brown between posterior ocellus and upper eye corner, isosceles, covered with long and mainly black pile. Ocellar triangle (Fig 7C) equilateral. Eyes dichoptic. Eye pile as long as scape, pale yellow.

Thorax: Scutum and scutellum black with bronze lustre, except pale yellow postpronotum and posterior rim of scutellum, as well as light brown transverse suture and postalar callus; predominantly shiny, but pospronotum and adjacent anterior areas with white microtrichia. Pleurae shiny without microtrichia. Wing hyaline, with brown veins and dense, light brown microtrichia (except in proximal 2/3 of br cell and above spurious vein with reduced microtrichia). Halter light brown. Femora brown, except usually with paler apex; pro- and mesotibiae completely orange-brown or vague shading subapically, metatibia orange-brown basally and apically; colour of tarsi varies (usually pro- and mesotarsi with two apical tarsomeres darkened dorsally and metatarsus dorsally darkened, the rest orange-brown). Metafemur thickened and straight (Fig 12A). Legs with pale yellow pile, except for some short black pile dorsally on tarsi.

Abdomen: Tergites predominately brown; except orange-yellow lateral spots on tergite 2, lateral sides of tergites, transversal bands (can be indistinct on tergite 4) and posterior 1/4 of tergite 4; transversal bands usually covered with white microtrichia. Pilosity pale yellow laterally on tergites, also whole of tergite 1 and most of tergite 4, as well as on orange transversal bands; black short adpressed pile cover posterior half of tergite 2 and dark areas of tergite 3. Sternites yellowish, covered with long pale yellow pile.

Male genitalia (Fig 13): Anterior lobe of surstyle with convex ventral margin (Fig 13A: a); median part of surstyle with two inner thorns (Fig 13B: t); posterior lobe of surstyle broad, short and triangular (Fig 13A: p). Hypandrium narrow, with unfolded thecal ridge (Fig 13C: r).

Female. Similar to the male except for normal sexual dimorphism (Figs 7B, 7E and 12B) and for the following characteristics: basoflagellomere 1.5–1.7 times as long as wide, with small, narrow, furrow-like fossette; frons and vertex without microtrichia except for narrow line along eye margin.

#### *Merodon melanocerus* Bezzi, 1915 (Figs 3, 9A–9F, 12C and 12D)

Holotype. ♀, *Merodon melanocerus* Bezzi, 1915: 101, RSA, Piet Retief Transvaal, 25 September 1903, R. Crawshay (sweep net), (BMNH).

Other material: RSA, Port St. John, 1♂, October 1916, (NMSA), 1♂, (NMNL), H. H. Swiny (sweep net), det. v. Doesburg as *Lampetia melanocera* Bezzi, (08693); Port St. John, Pondoland, 1♀, 6–25 February 1924, R. E. Turner (sweep net), (BMNH) (specimen bore labels indicating it as 'holotype' of *'M*. *marginatus'*—an undescribed species so designated by F. M. Hull); RSA, Natal, 75 km WSW Estcourt, Cathedral Peaks, 1860m, 2♂♂, 7–31 December 1979, S. & J. Peck (sweep net), (ZMUC) (515979, 515988); RSA, KwaZulu-Natal, Vryheid Nature Reserve, 27^o^ 45' S, 30^o^ 46' E, 1300m, 1♀, 2 December 1999, T. Dikow (sweep net), (NMSA) (64705); RSA, Drakensberg Mountains, Cathedral Peak, blue pools, 28^o^ 56' 45.14" S, 29^o^ 12' 02.72" E, 7.12.2012, 2♂♂, 1♀, 7 December 2012, A. Vujić (sweep net), (FSUNS) (G2193, G2197, G2201). RSA, Gilboa Mountain, -29°17'16.67" S, 30°17'35.71" E, 6♂♂, 3♀♀, 22–23 December 2016, A. Vujić, S. Radenković, N. Veličković & T. Petanidou (sweep net), (CEUA, FSUNS & MZH) (ZA3_046, ZA3_047, ZA3_048, ZA3_051, ZA3_053, ZA3_054, ZA3_049, ZA3_050, ZA3_052)

Range (Fig 1). Species is distributed in southeastern part of RSA. *Flight period*: February and July to December.

Diagnosis. This species is very similar to *Merodon drakonis* sp. n. because of the short basoflagellomere (almost as long as wide) and tergite 2 with reddish-orange lateral spots, as well as conspicuous gray-green microtrichia on pleurae. The main differences in males are in the shapes of genitalia: posterior lobe of surstyle triangular, with apex pointed towards tip of hypandrium (Fig 3A: p); ventral margin of anterior lobe of surstyle almost straight (Fig 3: a); anterior lobe of surstyle with large inner thorn (Fig 3B: at). In females, the distance between the posterior ocellus and upper eye corner in *M*. *melanocerus* is larger than the distance between ocelli (Fig 9D); in *M*. *drakonis* sp. n., this distance is smaller (Fig 9G). Based on genetic data and characteristics of the male genitalia (Fig 10), the most closely related species to *M*. *melanocerus* is *M*. *capensis* (see diagnosis for *M*. *capensis*).

Description. Male. Head (Fig 9A, 9C and 9E): Antenna (Fig 9E) dark-brown, basoflagellomere 1.0–1.1 times as long as wide, 1.1 times longer than pedicel; arista about 2.2 times longer than basoflagellomere. Vertical triangle (Fig 9C) isosceles, 2.5 times longer than eye contiguity, predominately covered with long black pile, except pale yellow pile at anterior and posterior ends. Ocellar triangle (Fig 9C) equilateral. Eye contiguity about 10 ommatidia long. Eye pile as long as scape, mostly pale yellow, except for some black pile on apical 1/6.

Thorax: Scutum black with bronze lustre and indistinct whitish microtrichia anteriorly that turn into three longitudinal stripes (one narrow medially and two wide submedially ending as spots at the level of the transverse suture); scutellum black, but in some specimens with brown posterior rim. Microtrichia of pleurae conspicuous, the most developed on katepisternum, gray-green in colour. Halter brown. Legs dark brown-black, except for paler knees, and sometimes proximal 1/3 dorsally on tibiae. Metafemur (Fig 12C) slightly thickened and curved, with short and adpressed hairs in apical 1/3. Legs covered with pale yellow pile.

Abdomen: Tergites 2–4 black, with distinct white transverse band of microtrichia interrupted in the middle (in some specimens lateral sides of tergites and ground colour of transverse bands also orange-brown); tergite 2 with orange antero-lateral spots; pile on tergites mainly erect and yellow on lateral sides, but tergites 2–4 medially with black adpressed pile. Sternites brown, but in some specimens sternite 2 and anterior margin of sternite 3 can be paler.

Male genitalia (Fig 3): Ventral margin of anterior lobe of surstyle almost straight (Fig 3A: a); anterior lobe of surstyle with large inner thorn (Fig 3B: at); median part of surstyle with small inner thorn (Fig 3B: t); posterior lobe of surstyle triangular, with apex pointed towards tip of hypandrium (Fig 3A: p). Hypandrium relatively narrow, with smooth thecal ridge (Fig 3C: r).

Female. Similar to the male except for normal sexual dimorphism and for the following characteristics: frons without microtrichia except narrow line along eye margin and microtrichose area at antennal level; on vertex, distance between posterior ocellus and upper eye corner larger than distance between ocelli.

#### Key for species of the *Merodon melanocerus* subgroup

Males

1. Eyes dichoptic (Fig 7C); basoflagellomere elongated, 1.5 times as long as wide (Fig 7D); male genitalia: posterior lobe of surstyle short, broad, triangular (Fig 13A: p)................................................................................. *Merodon flavocerus* Hurkmans sp. n.

- Eyes holoptic (as in Fig 9C); basoflagellomere short, 1.1 times as long as wide (as in Fig 9E); male genitalia: posterior lobe of surstyle with hook-like apex (as in Fig 3A: p).......................................................................................................................................... 2

2. Tergite 2 without large, distinct, orange lateral spots....................................................... 3

- Tergite 2 with large, distinct, orange lateral spots.............................................................. 4

3. Body hairs as usuall. Hypandrium with folded thecal ridge (Fig 2C: r), but without subapical lamellas and lateral wings.............................. *Merodon capensis* Hurkmans sp. n.

- Body hairs conspicuously thick, especially on vertical triangle and scutum. Hypandrium with smooth thecal ridge (Fig 8C), well-developed subapical lamellas (Fig 8C and 8D: s) and lateral wings (Fig 8C and 8D: w)................... *Merodon commutabilis* Radenković et Vujić sp. n.

4. Microtrichia on scutum well developed, including longitudinal stripe laterally on intraalar area till level of posterior end of postalar callus. Anterior lobe of surstyle with convex ventral margin (Fig 11A: a); hypandrium broad in apical fourth (Fig 11C), with oval subapical lamella (Fig 11C and 11D: s) and small lateral wings (Fig 11C and 11D: w)......................

........................................................................ *Merodon drakonis* Vujić et Radenković sp. n.

- Microtrichia on scutum indistinct, without microtrihose longitudinal stripe on intraalar area. Anterior surstyle lobe with almost straight ventral margin (Fig 3A: a) and large inner thorn (Fig 3B: at); hypandrium (Fig 3C) narrow in apical fourth, without subapical lamellas and lateral wings............................................... *Merodon melanocerus* Bezzi, 1915

Females

1. Tergite 2 black, at least laterally……......................................................................... 2

- Tergite 2 with large, distinct, orange lateral spots extending along lateral sides............... 3

2. Legs partly pale, at least pro- and mesotibiae at both ends, and basal two or three tarsomeres of pro- and mesotarsi; scutum with stripe of black pile between wing bases, the rest covered with pale yellow pile.................................. *Merodon capensis* Hurkmans sp. n.

- Legs black, sometimes metatarsi brown dorsally; scutum pilosity variable, can be covered with pale yellow or mixed black and pale yellow pile............................................................

................................................................ *Merodon commutabilis* Radenković et Vujić sp. n.

3. Basoflagellomere elongated, more than 1.5 times as long as wide (Fig 7E).......................

..................................................................................... *Merodon flavocerus* Hurkmans sp. n.

- Basoflagellomere short, 1.1 times as long as wide (as in Fig 9F)...................................... 4

4. Frons shiny, almost without microtrichia; distance between posterior ocelli and upper eye corner larger than distance between ocelli (Fig 9D)..........................................................

....................................................................................... *Merodon melanocerus* Bezzi, 1915

- Frons with lateral triangular microtrichose areas; distance between posterior ocelli and upper eye corner less than distance between ocelli (Fig 9G)....................................................

...................................................................... *Merodon drakonis* Vujić et Radenković sp. n.

### Molecular evidence of species delimitation within the *Merodon melanocerus* subgroup

The molecular analysis included 23 specimens from the *Merodon melanocerus* subgroup. Amplification of both the 3’- and 5’- end of the COI gene was successful for all individuals. The dataset containing the COI 5’ region sequences had a final length of 639 bp. The final length of the dataset containing the COI 3’ region sequences was 657 bp. A combined dataset of the 3’ and 5’ COI regions was used for the phylogenetic analyses.

**Fig 14:**
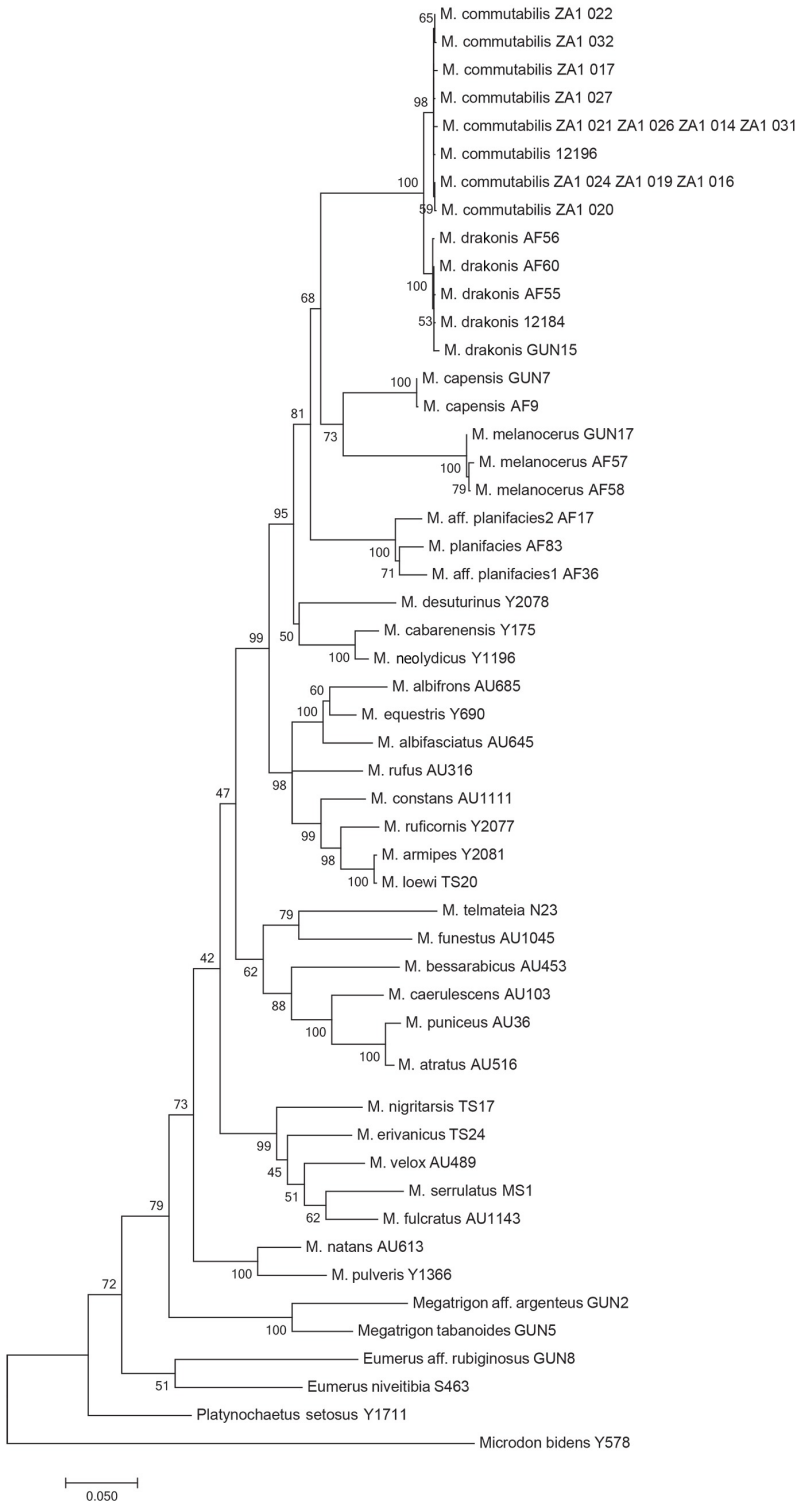
Maximum likelihood tree based on analysis of combined 3'- and 5'- fragments of the COI gene. The tree is drawn to scale, with branch lengths measured in the number of substitutions per site.

All obtained phylogenetic trees (ML, BI and MP) depict that the *Merodon melanocerus* subgroup belongs to the monophyletic *Merodon desuturinus* group (Figs 10 and 14 and S1 Fig). Inclusion of additional specimens from the genus *Merodon* in the phylogenetic trees allowed us to reveal four lineages (putative subgenera) within the *Merodon* genus; three previously established ones, *albifrons*+*desuturinus*, *aureus* (sensu lato) and *avidus*-*nigritarsis*, and one new lineage named *natans* (Fig 10). Within the *M*. *desuturinus* group, we observed clear differentiation of a Palaearctic lineage consisting of three species (*M*. *desuturinus*, *M*. *cabarenensis* and *M*. *neolydicus*) and two Afrotropical lineages consisting of the *M*. *planifacies* and the *M*. *melanocerus* subgroups, respectively. The positions of these three lineages within the *M*. *desuturinus* group differed on the MP tree compared to the ML and BI trees (these latter two exhibited the same topology). Based on the MP tree topology, the most divergent lineage within the group is the *M*. *planifacies* subgroup (S1 Fig). Unexpectedly, based on MP, the Palaearctic lineage was closer to the *M*. *melanocerus* subgroup that comprises specimens found exclusively in South Africa. In contrast, based on the ML and BI trees, the most divergent lineage was the Palaearctic one, and the *M*. *planifacies* and *M*. *melanocerus* subgroups were shown to be closely related.

The molecular data confirmed delineation of four species within the *M*. *melanocerus* subgroup: *M*. *melanocerus*, *M*. *capensis* sp. n., *M*. *drakonis* sp. n. and *M*. *commutabilis* sp. n. None of the specimens showed shared haplotypes among different species. According to the phylogenetic trees, within the *M*. *melanocerus* lineage, *M*. *drakonis* sp. n. and *M*. *commutabilis* sp. n. are genetically closely-related species and form one cluster, whereas *M*. *melanocerus* and *M*. *capensis* sp. n. are more divergent and constitute another cluster. Genetic relatedness revealed by molecular analyses also reflected relationships defined according to the structure of male genitalia.

## Discussion

The genus *Merodon* can be classified into about 20 monophyletic species groups, half of which were treated by Hurkmans [5] in his monograph. The majority of *Merodon* species are present in Eastern Europe and Asia Minor, which is associated with the high diversity of bulb species (their larval host plants) in these regions. Out of a total of ca. 160 species, less than 10 are known from the Afrotropical region. Only two species groups, *aureus* and *desuturinus*, have representatives in the Afrotropical region as well as in the Palaearctic.

We found that the *desuturinus* group consists of two clearly separate lineages based on both adult morphological and molecular data. The Palaearctic lineage includes four endemo-relicts: *M*. *cabanerensis* known only from a restricted highlands in central Spain and Morocco, *M*. *desuturinus* localized on high mountains in the Balkans, *M*. *murorum* from North-West Africa and *M*. *neolydicus* present in several countries in the Eastern Mediterranean (Greece, Turkey, Syria, Lebanon, Israel). Milankov et al. [26] observed low genetic variation in the species *M*. *desuturinus* and attributed this to the putative small sizes of the spatially isolated populations and its probable narrow ecological niche associated with possible historical events, especially genetic bottlenecks. This species occurs in Pleistocene refugia (high mountains of the Balkan Peninsula) and, together with the other three geographically-isolated Mediterranean taxa, can be considered oro-Mediterranean relicts.

The Afrotropical lineage comprises besides species *M*. *cuthbertsoni*, also two subgroups (*M*. *melanocerus* and *M*. *planifacies*). Contrary to all taxa of the *desuturinus* group, which have clear diagnostic adult morphological features, some members within the *planifacies* subgroup can only be delimited by genetic data. It is interesting that *M*. *cuthbertsoni*, a species from Zimbabwe (Southern Africa), also shares some distinct morphological characters with the South European endemo-relict, *M*. *desuturinus*. Unfortunately, there is no genetic data available for this South African endemic due to the fact that this species is only known from one specimen dated from 1925.

The *Merodon melanocerus* subgroup consists of five closely-related, though clearly morphologically and genetically separated, species distributed in the southeastern part of RSA. The most divergent species is *M*. *flavocerus* with several autapomorphic characters (basoflagellomere of antenna elongate and paler, katepisternum shiny, male eyes dichoptic, posterior lobe of surstyle shortened). Its distribution is restricted to the southern part of the Cape region. All records of this species date from the 20th century and, consequently, genetic data are missing. Additional species with an allopatric distribution is *M*. *capensis* sp. n., localized in the Western Cape Province. It is the most closely related to *M*. *melanocerus* species according to genetic data and features of male genitalia, but clearly different in external morphology. Structure of male genitalia represents the most conservative and stable morphological characters in hoverflies, important for species delimitation, but at the same time valuable for the revealing phylogenetic relationships. Resemblance in its shape reflects taxa relatedness [52, 53]. Another genetically close species, *M*. *commutabilis* sp. n. and *M*. *drakonis* sp. n., are also very similar in terms of structure of the male genitalia. During fieldwork, we observed that they appeared together, both sympatrically and synchronically, at some localities. Their distribution, and that of *M*. *melanocerus*, is associated with the southeastern part of RSA. Based on general external morphology, like the presence or absence of distinct orange lateral spots on tergite 2, less related species show greater resemblance to each other; for example *M*. *drakonis* sp. n. looks like *M*. *melanocerus*, or *M*. *capensis* sp. n. as *M*. *commutabilis* sp. n. However, there is no doubt that genetic data and structure of the male genitalia better reflect the true relationships than overall appearance, which often depends on habitat or other factors.

It is postulated that *Merodon* larvae all develop in the underground bulbs and rhizomes of geophytes (Amaryllidaceae, Iridaceae and Hyacinthaceae) or in the surrounding soil [5], based on data from eight species with described immature stages and three additional species whose larvae have been reared but are yet to be described (see review in [3, 4, 54]). Larvae of members of the *Merodon desuturinus* group are still undescribed but, according to field observations and unpublished data, they most probably also develop in plants of family Hyacinthaceae. Adults of *M*. *desuturinus* visit flowers of *Ornithogalum* and *Scilla* [26], and immature stages of the *M*. *planifacies* subgroup have been recorded in *Merwilla* bulbs. Family Hyacinthaceae comprises about 700–900 species, mainly distributed in Africa, Europe and SW Asia, with a single small genus in South America [55]. This distributional pattern suggests that diversification of this family began when North America was already clearly separated from Eurasia. Absence of the genus *Merodon* on the American continent can thus be linked to historical events in the Hyacinthaceae. However, the greatest diversity of Hyacinthaceae is found in South Africa [56], which is not in congruence with the exceptionally high diversity of *Merodon* in the Eastern Mediterranean. Migration of bulb species from southern Africa to northern Africa and Eurasia was possible during the late Early Miocene (c. 19 Mya.) when the Gomphotherium landbridge was formed and closed the Eastern Mediterranean seaway, giving rise to free exchange of flora and fauna between Africa and Eurasia [56, 57]. We suppose that diversification in the *M*. *desuturinus* group most probably happened much later, during fundamental shifts in African climate. Generally, the genus *Merodon* prefers warm, dry, open habitats with numerous bulb specimens. During the Pliocene-Pleistocene epoch, favorable conditions for *Merodon* species (increased aridity and open grasslands) in Africa [58] most probably allowed faunal transitions but, in the case of the *Merodon desuturins* group, this occurred in the opposite direction to the northward trajectory of the Hyacinthaceae; from the eastern Mediterranean (including SW Asia), one lineage migrated to South Africa and another to the western Palaearctic.

Separate position of the *desuturinus* group were revealed in earlier papers of Mengual et al. [13] and Milankov et al. [26], although each included only one species of the group (*M*. *cabanerensis* and *M*. *desuturinus*, respectively) in their analyses. Here, monophyly of the *desuturinus* group is confirmed (with an additional eight species analysed in relation to 27 other *Merodon* taxa), as well as its closest relationship to the *albifrons* group. Placement of the *melanocerus* subgroup on the obtained phylogenetic trees was variable. However, the MP tree showed a closer relationship of the *M*. *melanocerus* subgroup to species from the Palaearctic than to the *M*. *planifacies* subgroup which belongs to the Afrotropical lineage. In contrast, parametric ML and BI trees, commonly used to asses phylogeny, showed that the Afrotropical *M*. *melanocerus* and the *M*. *planifacies* subgoups are more closely related to each other than to Palaearctic species. To obtain a clearer picture of the relationships within the *desuturinus* group it will be necessary to obtain genetic data on *M*. *flavocerus*, *M*. *cuthbertsoni* and *M*. *stevensoni*, which is currently lacking since there are only old museum representatives available. We are planning future field investigations to fill missing gaps. Based on our phylogenetic analyses, we observe and name an additional lineage *natans*, in addition to three previously-established ones: *albifrons*+*desuturinus*, *aureus* (sensu lato) and *avidus*-*nigritarsis* described by Vujić et al. [9] and Šašić et al. [12]. These large lineages may reflect subgeneric divisions, but exploration of infrageneric structure and stability of these lineages will require a substantial expansion of taxa sampling for combined analyses of morphological and molecular data.

## Supporting information

S1 Table List of specimens used for molecular analysis and GenBank accession numbers for obtained sequences. (PDF)

S1 Fig Strict consensus tree based on four equally parsimonious trees from analysis of combined 3'- and 5'- fragments of the COI gene. Length 2040 steps, Consistency index (CI) 35, Retention index (RI) 66. Filled circles represent non-homoplasious changes and open circles are homoplasious changes.
(GIF)

## References

[1] Ståhls G, Vujic A, Perez-Banon C, Radenković S, Rojo S, Petanidou T. COI barcodes for identification of *Merodon* hoverflies (Diptera: Syrphidae) of Lesvos Island, Greece. Mol Ecol Res. 2009; 9: 1431–1438. 10.1111/j.1755-0998.2009.02592.x 21564929

[2] Ricarte A, Marcos-García MA, Rotheray GE. The early stages and life histories of three *Eumerus* and two *Merodon* species (Diptera: Syrphidae) from the Mediterranean region. Entomol Fenn. 2008; 19: 129–141.

[3] Andrić A, Šikoparija B, Obreht D, Đan M, Preradović J, Radenković S, et al DNA barcoding applied: Identification of the larva of *Merodon avidus* (Diptera: Syrphidae). Acta Entomol Mus Natl Pragae. 2014; 54(2): 741–757.

[4] Ricarte A, Souba-Dols GJ, Hauser M, Marcos-García MA. A review of the early stages and host plants of the genera *Eumerus* and *Merodon* (Diptera: Syrphidae), with new data on four species. PLoS ONE. 2017; 12(12): e0189852 10.1371/journal.pone.0189852 29261787 PMC5736194

[5] Hurkmans W. A monograph of *Merodon* (Diptera: Syrphidae). Part 1. Tijdschr Entomol. 1993; 136: 147–234.

[6] Milankov V, Ståhls G, Vujić A. Molecular diversity of populations of the *Merodon ruficornis* group on the Balkan Peninsula. J Zoolog Syst Evol Res. 2008a; 46: 143–152.

[7] Lj Francuski, Ludoški J, Vujić A, Milankov V. Wing geometric morphometric inferences on species delimitation and intraspecific divergent units in the *Merodon ruficornis* group (Diptera: Syrphidae) from the Balkan Peninsula. Zoolog Sci. 2009; 26: 301–308. 10.2108/zsj.26.301 19798925

[8] Lj Francuski, Ludoški J, Vujić A, Milankov V. Phenotypic evidence for hidden biodiversity in the *Merodon aureus* group (Diptera: Syrphidae) on the Balkan Peninsula: conservation implication. J Insect Conserv. 2011; 15: 379–388.

[9] Vujić A, Radenković S, Ståhls G, Ačanski J, Stefanović A, Veselić S, et al Systematics and taxonomy of the *ruficornis* group of genus *Merodon* Meigen (Diptera: Syrphidae). Syst Entomol. 2012; 37: 578–602.

[10] Vujić A, Radenković S, Likov L, Trifunov S, Nikolić T. Three new species of the *Merodon nigritarsis* group (Diptera: Syrphidae) from the Middle East. Zootaxa. 2013; 3640: 442–464. 26000427 10.11646/zootaxa.3640.3.7

[11] Vujić A, Radenković S, Ačanski J, Hayat R. Revision of the species of the *Merodon nanus* group (Diptera: Syrphidae) including three new species. Zootaxa. 2015; 4006(3): 439–462. 10.11646/zootaxa.4006.3.2 26623777

[12] Lj Šašić, Ačanski J, Vujić A, Ståhls G, Radenković S, Milić D, et al Molecular and morphological inference of three cryptic species within the *Merodon aureus*species group (Diptera: Syrphidae). PLoS ONE. 2016; 11(8): e0160001 10.1371/journal.pone.0160001 27532618 PMC4988715

[13] Mengual X, Ståhls G, Vujić A, Marcos-García M. Integrative taxonomy of Iberian *Merodon* species (Diptera: Syrphidae). Zootaxa. 2006; 1377: 1–26.

[14] Marcos-García MA, Vujić A, Mengual X. Revision of Iberian species of the genus *Merodon* Meigen, 1803 (Diptera: Syrphidae). Eur J Entomol. 2007; 104: 531–572.

[15] Marcos-García MA, Vujić A, Ricarte A, Ståhls G. Towards an integrated taxonomy of the *Merodon equestris* species complex (Diptera: Syrphidae) including description of a new species, with additional data on Iberian *Merodon*. Can Entomol. 2011; 143: 32–348.

[16] Petanidou T, Vujić A, Ellis WN. Hoverfly diversity (Diptera: Syrphidae) in a Mediterranean scrub community near Athens, Greece. Ann Soc Entomol Fr. 2011; 47: 168–175.

[17] Radenković S, Vujić A, Ståhls G, Pérez-Bañón C, Rojo S, Petanidou T, et al Three new cryptic species of the genus *Merodon* Meigen (Diptera: Syrphidae) from the island of Lesvos (Greece). Zootaxa. 2011; 2735: 35–56.

[18] Ricarte A, Nedeljković Z, Rotheray GE, Lyszkowski RM, Hancock EG, Watt K, et al Syrphidae (Diptera) from the Greek island of Lesvos, with description of two new species. Zootaxa. 2012; 3175: 1–23.

[19] Ståhls G, Vujic A, Petanidou T, Cardoso P, Radenković S, Ačanski J, et al Phylogeographic patterns of *Merodon* hoverflies in the Eastern Mediterranean region: revealing connections and barriers. Ecol Evol. 2016; 6(7): 2226–2245. 10.1002/ece3.2021 27069578 PMC4782255

[20] Vujić A, Pérez-Bañón C, Radenković S, Ståhls G, Rojo S, Petanidou T, et al Two new species of genus *Merodon* Meigen, 1803 (Syrphidae: Diptera) from the island of Lesvos (Greece), in the eastern Mediterranean. Ann Soc Entomol Fr. 2007; 43(3): 319–326.

[21] Vujić A, Marcos-García MA, Sarıbıyık S, Ricarte A. New data for the *Merodon* Meigen 1803 fauna (Diptera: Syrphidae) of Turkey including a new species description and status changes in several taxa. Ann Soc Entomol Fr. 2011; 47: 78–88.

[22] Pape T, Thompson FC. Systema Dipterorum, Version 1.5. Available at: http://www.diptera.org/; 2013.

[23] Manning JC, Goldblatt P, Snijman D. The Color Encyclopedia of Cape Bulbs Timber Press, Cambridge, 486 2002.

[24] Vujić A, Šimić S, Radenković S. *Merodon desuturinus*, a new hoverfly (Diptera: Syrphidae) from the mountain Kopaonik (Serbia). Ekologija. 1995; 30: 65–70.

[25] Vujić A, Radenković S, Likov L. Revision of the Palaearctic species of the *Merodon desuturinus* group (Diptera, Syrphidae). ZooKeys. 2018; 771: 105–138. 10.3897/zookeys.771.20481 PMC6043631 30008578

[26] Milankov V, Ståhls G, Vujić A. Genetic characterization of the Balkan endemic species, *Merodon desuturinus* (Diptera: Syrphidae). Eur J Entomol. 2008b; 105: 197–204.

[27] Jordaens K, Goergen G, Virgilio M, Backeljau T, Vokaer A, De Meyer M. DNA barcoding to improve the taxonomy of the Afrotropical hoverflies (Insecta: Diptera: Syrphidae). PLoS ONE. 2015; 10(10): e0140264 10.1371/journal.pone.0140264 26473612 PMC4608823

[28] Nedeljković Z, Ačanski J, Đan M, Obreht Vidaković D, Ricarte A, Vujić A. An integrated approach to delimiting species borders in the genus *Chrysotoxum* Meigen, 1803 (Diptera: Syrphidae), with description of two new species. Contrib Zool. 2015; 84(4): 285–304.

[29] Hebert PDN, Cywinska A, Ball SL, deWaard JR. Biological identifications through DNA barcodes. Proc R Soc Lond B Biol Sci. 2003a; 270: 313–322. 10.1098/rspb.2002.2218 PMC1691236 12614582

[30] Hebert PDN, Ratnasingham S, deWaard JR. Barcoding animal life: cytochrome c oxidase subunit 1 divergences among closely related species. Proc R Soc Lond B Biol Sci. 2003b; 270 (Suppl 1): S96–S99. 10.1098/rsbl.2003.0025 PMC1698023 12952648

[31] Tautz D, Arctander P, Minelli A, Thomas RH, Vogler AP. A plea for DNA taxonomy. Trends Ecol Evol. 2003; 18(2): 71–74.

[32] Gaston KJ, O’Neill MA. Automated species identification: why not? Philos Trans R Soc Lond B Biol Sci. 2004; 359: 655–667. 10.1098/rstb.2003.1442 15253351 PMC1693351

[33] Ratnasingham S, Hebert PDN. BOLD: The Barcode of Life Data System. Mol Ecol Res. 2007; 7: 355–364. Available at: http://www.barcoding.life.org. 10.1111/j.1471-8286.2007.01678.x PMC1890991 18784790

[34] Milankov V, Stamenković J, Ludoški J, Ståhls G, Vujić A. Diagnostic molecular markers and the genetic relationships among tree species of the *Cheilosia canicularis* group (Diptera: Syrphidae). Eur J Entomol. 2005; 102: 125–131.

[35] Ståhls G, Vujić A, Milankov V. *Cheilosia vernalis* complex: molecular and morphological variability (Diptera: Syrphidae). Ann Zool Fenn. 2008; 45(2): 149–159.

[36] Hijmans RJ, Guarino L, Mathur P. DIVA-GIS.Vsn. 7.5. A geographic information system for the analysis of species distribution data. Available at: http://www.diva-gis.org; 2012. (accessed 1 November 2016).

[37] Thompson FC. Key to the genera of the flower flies (Diptera: Syrphidae) of the Neotropical Region including descriptions of new genera and species and a glossary of taxonomic terms. Contrib Am Entomol. 1999; 3: 321–378.

[38] Doczkal D, Pape T. *Lyneborgimyia magnifica* gen. et sp. n. (Diptera: Syrphidae) from Tanzania, with a phylogenetic analysis of the Eumerini using new morphological characters. Syst Entomol. 2009; 34: 559–573.

[39] Hadley A. CombineZ. Version 5.3. Available at: http://www.hadleyweb.pwp.blueyonder.co.uk; 2006. (accessed 10 June 2016).

[40] Chen H, Rangasamy M, Tan SY, Wang H, Siegfried BD. Evaluation of five methods for total DNA extraction from Western corn rootworm beetles. PLoS ONE. 2010; 5(8): e11963 10.1371/journal.pone.0011963 20730102 PMC2921343

[41] Simon C, Frati F, Beckenbach AT, Crespi B, Liu H, Flook P. Evolution, weighting, and phylogenetic utility of mitochondrial gene sequences and a compilation of conserved polymerase chain reaction primers. Ann Entomol Soc Am. 1994; 87: 651–701.

[42] Folmer O, Black M, Hoeh W, Lutz R, Vrijenhoek R. DNA primers for amplification of mitochondrial cytochrome c oxidase subunit I from diverse metazoan invertebrates. Mol Marine Biol Biotechnol. 1994; 3(5): 294–299. 7881515

[43] Thompson JD, Higgins DG, Gibson TJ. Clustal W: improving the sensitivity of progressive multiple sequence alignment through sequence weighting, position-specific gap penalties and weigh matrix choice. Nucleic Acids Res. 1994; 22: 4673–4680. 7984417 10.1093/nar/22.22.4673 PMC308517

[44] Hall TA. BioEdit: a user-friendly biological sequence alignment editor and analysis program for Windows 95/98/NT. Nucleic Acids Symp Ser. 1999; 41: 95–98.

[45] Xia X. DAMBE5: A comprehensive software package for data analysis in molecular biology and evolution. Mol Biol Evol. 2013; 30: 1720–1728. 10.1093/molbev/mst064 23564938 PMC3684854

[46] Kumar S, Stecher G, Tamura K. MEGA7. Molecular Evolutionary Genetics Analysis. Version 7.0. Mol Biol Evol. 2015; 33(7): 1870–1874. 10.1093/molbev/msw054 PMC8210823 27004904

[47] Ronquist F, Teslenko M, van der Mark P, Ayres DL, Darling A, Höhna S, Larget B, et al MrBayes 3.2: efficient Bayesian phylogenetic inference and model choice across a large model space. Syst Biol. 2012; 61: 539–542. 10.1093/sysbio/sys029 22357727 PMC3329765

[48] Rambaut A, Suchard MA, Xie D, Drummond AJ. Tracer v1.6. 2014. Available at: http://beast.bio.ed.ac.uk/Tracer. (accessed on 24 September 2014).

[49] Rambaut, A. 2014. FigTree–The Figure Drawing Tool, version 1.4.2. Institute of Evolutionary Biology, University of Edinburgh. Available at: http://tree.bio.ed.ac.uk/figtree/ (accessed on 24 September 2014).

[50] Goloboff PA. NONA computer program. Ver. 2.0. Tucuman, Argentina: Published by the author; 1999.

[51] Nixon KC. WinClada ver. 1.00. 08. Ithaca, New York: Published by the author; 2002.

[52] Hippa H, Ståhls G. Morphological characters of adult Syrphidae: descriptions and phylogenetic utility Finnish Zoological and Botanical Publishing Board, Helsinki; 2005.

[53] Rotheray GE, Gilbert F. The natural history of hoverflies Ceredigion: Forrest text; 2011.

[54] Preradović J, Andrić A, Radenković S, Šašić Zorić Lj, Pérez-Bañón C, Campoy A, et al Pupal stages of three species of the phytophagous genus *Merodon* Meigen (Diptera: Syrphidae). Zootaxa. 2018; 4420(2): 229–242. 10.11646/zootaxa.4420.2.5 30313544

[55] Martínez-Azorín M, Crespo MB, Juan A, Fay MF. Molecular phylogenetics of subfamily Ornithogaloideae (Hyacinthaceae) based on nuclear and plastid DNA regions, including a new taxonomic arrangement. Ann Bot. 2011; 107: 1–37. 10.1093/aob/mcq207 21163815 PMC3002468

[56] Pfosser MF, Speta F. From *Scilla* to *Charybdis*—is our voyage safer now? Plant Syst Evol. 2004; 246: 245–263.

[57] Rögl F. Peleogeographic considerations for Mediterranean and Paratethys seaways (Oligocene to Miocene). Ann Nat Hist Mus Wien. 1998; 99A: 279–310.

[58] De Menocal P. African climate change and faunal evolution during Pliocene- Pleistocene. Earth Planet Sci Lett. 2004; 220: 3–24.

