# Supplementary material for: Close relatives of Mediterranean endemo-relict hoverflies (Diptera, Syrphidae) in South Africa: Morphological and molecular evidence in the Merodon melanocerus subgroup
Source: PLoS One. 2018 Jul 20;13(7):e0200805. doi: 10.1371/journal.pone.0200805 (PMC6054422; doi:10.1371/journal.pone.0200805)
Supplement: S1 Table — (PDF) [file pone.0200805.s001.pdf]

Appendix S1. List of specimens used for molecular analysis and GenBank accession numbers for obtained sequences.

**Taxon**	**Laboratory code**	**GenBank accession number 3'COI**	**GenBank accession number 5'COI**
Merodon capensis Hurkmans sp. n.	GUN7	MG785716	MG785716
Merodon capensis Hurkmans sp. n.	AF9	MG785717	MG785717
Merodon commutabilis Radenković et Vujić sp. n.	ZA1_014	MG785708	MG785708
Merodon commutabilis Radenković et Vujić sp. n.	ZA1_016	MG785700	MG785700
Merodon commutabilis Radenković et Vujić sp. n.	ZA1_017	MG785702	MG785702
Merodon commutabilis Radenković et Vujić sp. n.	ZA1_019	MG785699	MG785699
Merodon commutabilis Radenković et Vujić sp. n.	ZA1_020	MG785701	MG785701
Merodon commutabilis Radenković et Vujić sp. n.	ZA1_021	MG785706	MG785706
Merodon commutabilis Radenković et Vujić sp. n.	ZA1_022	MG785704	MG785704
Merodon commutabilis Radenković et Vujić sp. n.	ZA1_024	MG785698	MG785698
Merodon commutabilis Radenković et Vujić sp. n.	ZA1_026	MG785707	MG785707
Merodon commutabilis Radenković et Vujić sp. n.	ZA1_027	MG785703	MG785703
Merodon commutabilis Radenković et Vujić sp. n.	ZA1_031	MG785709	MG785709
Merodon commutabilis Radenković et Vujić sp. n.	ZA1_032	MG785705	MG785705
Merodon commutabilis Radenković et Vujić sp. n.	12196	MG785710	MG785710
Merodon drakonis Vujić et Radenković sp. n.	AF55	MG785711	MG785711
Merodon drakonis Vujić et Radenković sp. n.	AF56	MG785712	MG785712
Merodon drakonis Vujić et Radenković sp. n.	AF60	MG785714	MG785714
Merodon drakonis Vujić et Radenković sp. n.	12184	MG785713	MG785713
Merodon drakonis Vujić et Radenković sp. n.	GUN15	MG785715	MG785715
Merodon melanocerus Bezzi, 1915	AF57	MG785718	MG785718
Merodon melanocerus Bezzi, 1915	AF58	MG785719	MG785719
Merodon melanocerus Bezzi, 1915	GUN17	MG785720	MG785720
Merodon planifacies Bezzi, 1915	AF83	MF497644	MF497688
Merodon aff. *planifacies* 1	AF17	MF497620	MF497664
Merodon aff. *planifacies* 2	AF36	MF497601	MF497645
Merodon cabanerensis Marcos-García, Vujić et Mengual, 2007	Y175	DQ386316	MH521953
Merodon desuturinus Vujić, Šimić et Radenković, 1995	Y2078	MH496003	LT882606
Merodon neolydicus Hurkmans et Vujić, 2018	Y1196	MH496004	MH496010
Merodon velox Paramonov, 1925	AU489	MH511638	LN995533
Merodon serrulatus Wiedemann, 1822	MS1	MH511641	MH511641

Merodon fulcratus Sack, 1913	AU1143	MH511642	MH511642
Merodon erivanicus Paramonov, 1925	TS24	MH511643	MH511643
Merodon nigritarsis Rondani, 1845	TS17	MH511644	MH511644
Merodon pulveris Vujić et Radenković, 2011	Y1366	MH496002	MH496007
Merodon bessarabicus Paramonov, 1924	AU453	MH511645	MH511645
Merodon atratus Oldenberg, 1919	AU516	KU365432	KU365496
Merodon puniceus Vujić, Radenković et Pérez-Bañón, 2011	AU36	MH511646	MH511646
Merodon caerulescens Loew, 1869	AU103	MH133981	MH133981
Merodon telmateia Hurkmans, 1987	N23	MH538385	MH538385
Merodon funestus Fabricius, 1794	AU1045	MH511647	MH511647
Merodon armipes Rondani, 1843	Y2081	MH496000	MH496012
Merodon loewi Goot, 1964	TS20	MH511648	MH511648
Merodon ruficornis Meigen, 1822	Y2077	MH496001	MH496011
Merodon constans Rossi, 1794	AU1111	MH511649	MH511649
Merodon equestris Fabricius, 1794	Y690*	EU431486	EU431486
Merodon albifasciatus Macquart, 1842	AU645	MH511650	MH511650
Merodon albifrons Meigen, 1822	AU685	MH511651	MH511651
Merodon rufus Meigen, 1838	AU316	MH511652	MH511652
Merodon natans Fabricius, 1794	AU613	MH511653	MH511653
Megatrigon tabanoides Doczkal, Radenković, Lyneborg, Pape, 2016	GUN5	KT157921	MH511640
Megatrigon aff. *argenteus*	GUN2	KT157920	MH511639
Platynochaetus setosus Fabricius, 1794	Y1711	KM224512	MH521922
Eumerus aff. *rubiginosus*	GUN8	MH511654	MH511654
Eumerus niveitibia Becker, 1921	S463	not submitted	MG604934
Microdon bidens Fabricius, 1805	Y578	HF547911	HF934120

*http//:id.luomus.fi/GJ.2774
