# Supplementary figures and images for: Close relatives of Mediterranean endemo-relict hoverflies (Diptera, Syrphidae) in South Africa: Morphological and molecular evidence in the Merodon melanocerus subgroup
Source: PLoS One. 2018 Jul 20;13(7):e0200805. doi: 10.1371/journal.pone.0200805 (PMC6054422; doi:10.1371/journal.pone.0200805)

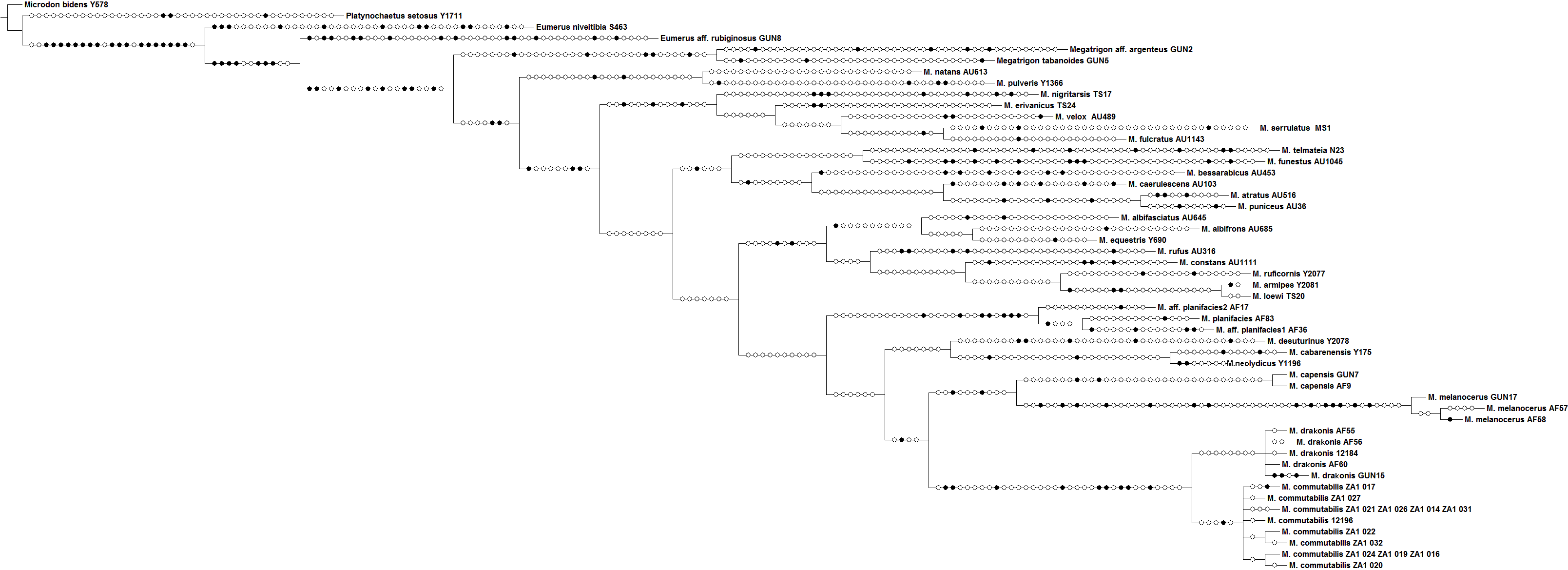

Supplement: S1 Fig — Length 2040 steps, Consistency index (CI) 35, Retention index (RI) 66. Filled circles represent non-homoplasious changes and open circles are homoplasious changes. (GIF) [file pone.0200805.s002.gif]
